# Investigation of biomarkers and associated molecular mechanism shared between colorectal cancer and lung adenocarcinoma

**DOI:** 10.1007/s12672-025-03240-5

**Published:** 2025-08-12

**Authors:** Kun Gao, Lianyang Yu, Lei Wang, Linlu Gao, Tingting Li, Jing Sun

**Affiliations:** 1 Department of Oncology, Zibo integrated Chinese and Western medicine hospital, ZiBo City, 255000 Shandong Province China; 2 Department of Pulmonary Diseases, Zibo integrated Chinese and Western medicine hospital, ZiBo City, 255000 Shandong Province China

**Keywords:** Colorectal cancer, Lung adenocarcinoma, Biomarkers, GO and KEGG pathway analysis, Immune infiltration, Validation analysis

## Abstract

**Background:**

The relationship between colorectal cancer (CRC) and lung adenocarcinoma (LUAD) has been acknowledged in recent years, yet the biomarkers and mechanisms underlying their interaction remain unclear. This study aimed to explore the genetic characteristics and molecular mechanisms shared by CRC and LUAD.

**Methods:**

To identify common differentially expressed genes (co-DEGs) between tumor and normal samples, we analyzed gene expression datasets for CRC and LUAD from the GEO and TCGA databases. Then, co-DEGs were further analyzed for enrichment. The survival analysis and protein-protein interaction (PPI) analysis were used to explored biomarkers for two diseases. Based on the identified biomarkers, the functional similarity, immune cell infiltration, and Gene Set Enrichment Analyses (GSEA) were performed. Moreover, the drug-gene interaction was predicted based on biomarkers. Finally, the qRT-PCR, Western blotting, and enzyme-linked immunosorbent assay (ELISA) to validate the expression of these biomarkers in CRC and LUAD based on cell lines and mice model.

**Results:**

We explored totally 3470 co-DEGs of tumor vs. normal based on CRC and LUAD dataset. K-M survival and PPI network analysis revealed five hub prognostic genes, including HSPA6, NOTCH3, PKP2, SMAD9, and GPD1L, as biomarkers for two diseases, with enrichment analysis showing that biomarkers like HSPA6 and SMAD9 were primarily associated with protein binding functions. Functional similarity analysis revealed that the five biomarkers exhibited a high degree of similarity in their biological roles, with NOTCH3 showing the highest similarity, further supported by immune infiltration analysis indicating a significant and strong positive correlation between NOTCH3 and Natural Killer cells. Finally, the expression investigation of five biomarkers based on cell lines and mice model validated the results of bioinformatics analysis.

**Conclusions:**

HSPA6, NOTCH3, PKP2, SMAD9, and GPD1L were five novel biomarkers for CRC and LUAD clinical diagnosis or treatment. HSPA6 and SMAD9 might take part in the progression of CRC and LUAD via protein binding function.

**Supplementary Information:**

The online version contains supplementary material available at 10.1007/s12672-025-03240-5.

## Background

Colorectal cancer (CRC), a malignant neoplasm developing from the epithelial cells of the colon or rectum, is the third most prevalent cancer in men and the second most prevalent cancer among women [1]. The development of CRC as a secondary primary cancer in individuals who have survived lung cancer has emerged as a critical factor influencing the prognosis of early-stage lung cancer patients [2]. Lung adenocarcinoma (LUAD) is the most prevalent sub-type of lung cancer, particularly among non-smoking females and younger patients [3]. CRC and LUAD rank among the most dangerous diseases due to their high incidence and mortality rates. Despite being situated in distinct organs and having diverse clinical features, these cancers exhibit shared molecular traits and pathways that could play crucial roles in their development.

A recent Mendelian randomization (MR) analysis proves that the overall lung cancer and CRC were dramatically related to the genetic level using MR methods including inverse variance weighted [4]. However, early-stage CRC and LUAD are often asymptomatic, which leads to delayed diagnosis and a poor prognosis for many patients [5]. Current diagnostic and prognostic approaches for these cancers are limited in their sensitivity and specificity, highlighting the urgent need for reliable biomarkers. Actually, the identification of common biomarkers involved in the progression of two diseases, as well as understanding their molecular mechanisms are crucial steps for the development of broad-spectrum diagnostic tools and targeted therapies [6]. It has been demonstrated that *KRAS* and *BRAF* are strongly linked to poor outcomes and resistance to targeted therapies in CRC and LUAD patients, often in conjunction with other mutant activated genes [7]. Additionally, certain biomarkers have been shown to activate mechanisms that recruit molecular agents. When targeted, these agents, along with the involvement of the tumor microenvironment (TME), can gradually accumulate to a critical level, facilitating tumor progression and leading to treatment failure [8]. A previous bioinformatics study by Dai et al. indicated that *CLU*, *SFTPD*, and *CCL18* may serve as key genes linked to lung-specific metastasis in CRC, with their protein levels detected in primary CRC cells, lung metastatic cells, serum, or tissues [9]. Furthermore, advances in high-throughput sequencing technologies and bioinformatics have enabled comprehensive genomic and transcriptomic profiling of various cancers [10]. These methods have enabled the discovery of crucial genes and pathways that play a role in tumor initiation and advancement [11]. However, most studies have focused on individual cancer types, with limited exploration of cross-cancer molecular signatures that play crucial roles in the metastatic progression of several cancer types [12]. Understanding the overlapping genetic and molecular mechanisms between CRC and LUAD could unveil novel biomarkers that are not only diagnostically relevant but also potentially targetable for therapeutic interventions.

In this study, we hoped to reveal common genes implicated in the progression of CRC and LUAD and to elucidate their molecular roles in these cancers. By leveraging integrative bioinformatics approaches, as well as validating findings in mice model and cell lines, we seek to contribute to the development of improved diagnostic and prognostic biomarkers that could enhance clinical outcomes for patients suffering from these malignancies.

## Materials and methods

### Microarray data and pre-processing

The Cancer Genome Atlas (TCGA) is a comprehensive database that collects the gene expression profiles in 33 cancer types [13]. The RNA-seq data and corresponding survival/clinical information of TCGA-CRC and TCGA-LUAD were obtained from Pan-Cancer Atlas Hub in UCSC Xene database (https://xenabrowser.net) as the training dataset. Based on the target clinical information (cancer_name: COAD and READ), a total of 383 CRC samples (371 samples with survival and clinical information) and 51 normal tissue samples were enrolled in current TCGA-CRC dataset, while totally 510 LUAD samples (490 samples accompanied by survival and clinical data) and 58 normal tissue samples were enrolled in current TCGA-LUAD dataset. Additionally, the microarray datasets GSE39582 and GSE30219 were obtained from the Gene Expression Omnibus (GEO) database for external validation. GSE39582 included 566 CRC samples and 19 normal samples, while GSE30219 comprised 272 LUAD samples and 14 normal samples. The data was generated on GPL570 Affymetrix Human Genome U133 Plus 2.0 Array. All these two GEO datasets were based on the platform of GPL570 (HG-U133_Plus_2) Affymetrix Human Genome U133 Plus 2.0 Array.

### The common-differentially expressed genes (co-DEGs) analysis

The limma package (version: 3.34.7) [14] in R software was employed to explore the DEGs between tumor and normal samples in both TCGA-CRC dataset and TCGA-LUAD dataset, followed by the corresponding P values and log fold change (FC) values of genes obtained. Genes with Benjamini & Hochberg (BH) adjusted p-value of less than 0.05 and an absolute|log2 FC| greater than 0.5 were set as the criteria for DEG selection. The result was visualized by using ggplot2 and pheatmap package in R software [15]. In addition, a Venn diagram analysis was performed to identify the co-DEGs by intersecting the DEGs from CRC vs. control and LUAD vs. control by using VennDiagram package in R [16].

### Enrichment analysis on co-DEGs

Gene Ontology (GO) and Kyoto Encyclopedia of Genes and Genomes (KEGG) analyses uncovered critical biological functions and pathways linked to the target genes [17]. In this study, GO functional analysis and KEGG pathway enrichment were conducted on DEGs using DAVID software [18]. The GO analysis covered biological processes (BP), cellular components (CC), and molecular functions (MF). A p-value < 0.05 and a count ≥ 2 were set as the thresholds. The top 10 GO and KEGG enrichment results were visualized using bubble plots.

### Prognostic genes investigation

All tumor samples in two training datasets were categorized into high and low expression groups based on the median expression levels of the co-DEGs, followed by the Kaplan-Meier (K-M) analysis using survival package in R software [19]. *P* < 0.001 was considered to be significantly associated with the survival of sepsis. Then, the common prognostic genes (hub genes) in two datasets were revealed by using VENN plot analysis.

### Protein-protein interaction (PPI) network investigation

STRING (version: 11.5) was used to predict the interactive relation associated with hug genes with confidence as 0.15 [20]. Subsequently, the results of the PPI network were analyzed further, and the hub genes identified within the PPI network were ranked by their network features using the CytoHubba plugin in Cytoscape [21]. CytoHubba offers 12 different topological analysis methods for network evaluation, including Maximal Clique Centrality (MCC), Density of Maximum Neighborhood Component (DMNC), Maximum Neighborhood Component (MNC), Degree Centrality (Degree), Edge Percolated Component (EPC), and seven centrality measures (Bottleneck, EcCentricity, Closeness, Radiality, Betweenness, Clustering Coefficient, and Stress) that are based on shortest path calculations. After screening and ranking the core nodes in the network, the core nodes (the intersection of TOP10 genes in topological analysis methods) were revealed as biomarkers in current study. Finally, a functional similarity analysis was performed using the GOSemSim package to assess the relatedness of the five biomarkers based on their associated GO terms. This analysis is based on the assumption that functionally similar gene products are likely to be clustered together within the GO tree (goSim, mgoSim, geneSim and clusterSim).

### The expression and cox regression analysis based on biomarkers

The expression levels of the biomarkers were extracted from the CRC/LUAD training and validation datasets. Based on the grouping information of each dataset, the ggplot2 package in R was employed to explore the expression of the biomarkers in the two datasets. The Wilcoxon test was used to evaluate the statistical significance of expression level differences between the groups. Univariate and multivariate regression analyses of biomarkers were performed by survival package in R (Version2.41-1, http://bioconductor.org/packages/survivalr/).

### Immune cell infiltration analysis among subtypes

Single-sample Gene Set Enrichment Analysis (ssGSEA) can quantitatively immune infiltration levels of 28 kinds of immune cells in tumor samples [22]. Based on ssGSEA, the relative abundance of tumor-infiltrating immune cells (TIICs) in each sample from the CRC/LUAD training dataset was estimated by calculating the enrichment fraction of each immune cell type. Wilcoxon’s signed-rank test was employed to examine differences in immune cell abundance between groups. In addition, the correlation analysis between significant TIICs and biomarkers CRC/LUAD training dataset was performed using Spearman [23]. The results were visualized by using ggplot2 package in R.

### Gene set enrichment analysis (GSEA)

Gene Set Enrichment Analysis (GSEA) is a powerful computational method that identifies statistically significant pathway or biological process in predefined gene set. The Pearson correlation coefficients of each biomarker and all genes were calculated based on the CRC/LUAD training dataset, and then the correlation coefficients were sorted from largest to smallest. Based on c2.cp.kegg.v7.4.symbols.gmt enrichment background in MSigDB database [24], the enrichment scores of each KEGG pathway in each biomarker were calculated and sorted using with BH adjusted *P* < 0.05 using clusterProfiler package in R software. The Top 10 of each enrichment results were listed according to the normalized enrichment score (NES).

### Drug-gene interaction prediction

The Comparative Toxicogenomics Database (CTD) is collection of the interactions between chemicals, genes and diseases [25]. Drugs associated with the disease were investigated using CTD with keywords (gene symbol) for each biomarker [26]. Subsequently, a drug-gene interaction network was created using Cytoscape software.

### The qRT-PCR analysis

To further investigate the expression of biomarkers (*HSPA6*, *NOTCH3*, *PKP2*, *SMAD9* and *GPD1L*) revealed in current study, a verification study was performed based on cultured CRC cell line SW480 (ATCC, Catalog Number: CCL-228™) vs. normal colon cell line NCM460 (INCELL, Catalog Number: M-H-460), as well as LUAD cell line A549 (ATCC, Catalog Number: CCL-185™) vs. normal lung cell line BEAS-2B (ATCC, Catalog Number: CRL-9609™). Briefly, SW480 and A549 cells were maintained in DMEM and RPMI-1640 (10% FBS), respectively, while NCM460 and BEAS-2B were maintained in specialized media (M-H and BEGM™). Cells were cultured at routine condition of 37 °C/5% CO_2_. After culture, total RNAs were extracted using TRIZOL reagent (Invitrogen, Catalog Number: 15596018), and reversely transcribed using RevertAidTM First Strand cDNA Synthesis Kit (Takara, Catalog Number: RR036A) in accordance with manufacturers’ instructions. The PCR was performed on ABI7500 (Applied Biosystems, U.S.A.). The detailed information for all primers used in current study were listed in Table 1. The PCR program included 95 °C for 5 min, 35 cycles of 95 °C for 30 s and 52 °C for 30s. The relative expression was calculated using the 2 ^−ΔΔCt^ method.

**Table 1:** The detailed information for all primers used in current study

Primer	Sequence(5’−3’)
HSPA6-F	TCTACTTCCCCGAGAATGCC
HSPA6-R	GTGGTGATGGTGTTGTCGTT
NOTCH3-F	GAGGGTTCCCAAGTGATCC
NOTCH3-R	CAGCTTCCTCTTGTCGTTTCT
PKP2-F	AAGGTGACAGTGCGTGTGA
PKP2-R	CAGGAGGAGGAGGTGGAAG
SMAD9-F	CGCCTGGCTATCCTTACCTC
SMAD9-R	GCTGAGAGGATGGCACATC
GPD1L-F	CTGGAAGAGGCTGAGAGGTT
GPD1L-R	CAGGTTGAGTGGAAGGGTTC
GAPDH-F	GAA GGT GAA GGT CGG AGT
GAPDH-R	GAA GAT GGT GAT GGG ATT TC

Notes: GAPDH, Glyceraldehyde 3-phosphate dehydrogenase

### CRC and LUAD mice model construction and sample collection

Experiments were conducted using six-week-old male BALB/c mice (SPF grade) obtained from Beijing Vital River Laboratory Animal Technology Co., Ltd. The mice were maintained in a controlled environment with a temperature of 22 ± 2 °C, relative humidity of 50 ± 10%, and a 12-hour light/dark cycle, with ad libitum access to food and water.

The CRC model was induced using chemical agents. The mice in experimental group (n = 5) were received intraperitoneal injections of 10 mg/kg Azoxymethane (AOM, Sigma-Aldrich, Catalog Number: A5486) once per week for two consecutive weeks. Subsequently, 2% Dextran sulfate sodium (DSS, MP Biomedicals, Catalog Number: 160110) solution was administered in drinking water for five days, followed by normal water, with this cycle repeated twice. The mice in control group (n = 5) received equivalent volumes of saline and normal water without DSS. After mice model construction, the weight of CRC mice, the stool form and fecal occult blood were observed and recorded. Disease activity index (DAI) of CRC mice was calculated according to the following formula: DAI =(weight loss rate score + stool form score + fecal occult blood score)/3 [27, 28].

The LUAD model was established using a xenograft method. The mice in experimental group (*n* = 5) were anesthetized with ether and subcutaneously injected with 5 × 10^6^ Lewis lung carcinoma cells (LLC, ATCC, Catalog Number: CRL-1642) in a 100 µL suspension into the right axilla. The mice in control group (*n* = 5) were received an equivalent volume of PBS under the same conditions. The growth of LUAD was measured by vernier caliper each week and the volume of tumor tissues were calculated.

During the experimental process, the survival time of the mice models were recorded. At the conclusion of the experiments, mice were euthanized via cervical dislocation, and both tumor and normal tissues were promptly harvested. A portion of the tissues was used for protein extraction, while another portion was used for serum sample collection. The samples from both the experimental and control groups were processed and analyzed to compare the expression levels of the biomarkers. All animal experiments were conducted in strict accordance with ethical standards for animal welfare, following the Guide for the Care and Use of Laboratory Animals (8th Edition, National Academies Press, 2011), and the experimental protocol was approved by the Ethics Committee of our hospital *(*Approval No. [2023] Lun Shen No. 16).

### Western blotting

Protein extraction from cell lysates was performed with RIPA buffer, which included inhibitors for proteases and phosphatases. Tissue samples were mixed with SDS loading buffer and heated for 10 min. Proteins (25 µg per lane) were separated using SDS-PAGE and subsequently transferred to polyvinylidene difluoride (PVDF) membranes (Millipore Corp, MA, USA). Following a 1-hour incubation with 5% BSA in TBST, the membranes were incubated with anti-HSPA6 antibody (1:1000, Abcam, Catalog Number: ab13492), anti-NOTCH3 antibody (1:1000, Cell Signaling Technology, Catalog Number: 2889 S), anti-SMAD9 antibody (1:1000, Thermo Fisher Scientific, Catalog Number: PA5-47403), anti-GPD1L antibody (1:1000, Santa Cruz Biotechnology, Catalog Number: sc-365415) at 4 °C overnight. The membranes were washed three times with TBST and then incubated with secondary antibody (HRP-conjugated, Jackson ImmunoResearch, Catalog Number: 111-035-144) diluted at 1:5000 for 1 h. Western blotting was performed with three biological replicates per group. Chemiluminescent signals were detected using the ChemiDoc MP system and quantified with Image J software.

### Enzyme-linked immunosorbent assay (ELISA) analysis

The *HSPA6*, *NOTCH3*, *PKP2*, *SMAD9* and *GPD1L* levels were determined using *HSPA6* ELISA Kit (Cloud-Clone Corp., Catalog Number: CEA836Mu), *NOTCH3* ELISA Kit (Cloud-Clone Corp., Catalog Number: CEA971Mu), *PKP2* ELISA Kit (MyBioSource, Catalog Number: MBS2804148), *SMAD9* ELISA Kit (Cloud-Clone Corp., Catalog Number: SEA109Mu) and *GPD1L* ELISA Kit (MyBioSource, Catalog Number: MBS2025114). Serum samples were prepared by centrifugation (1,500 × g, 10 min, 4 °C) and stored at −80 °C. For protein detection, samples were diluted at 1:5 (PBS with 1% BSA) and 50 µL sample was loaded per well. Samples were incubated with primary antibodies overnight at 4 °C, followed by biotinylated detection antibody. Staining was performed with Streptavidin-HRP (1:10,000, 100 µL/well). Absorbance at 450 nm was measured using an enzyme-linked immunosorbent assay reader (PerkinElmer, Waltham, MA, United States).

### Statistical analysis

Data analysis was performed with GraphPad Prism (version 8.0.1). An unpaired two-tailed t-test was used to compare differences between groups. Results were presented as mean ± SD, with significance defined as *P* < 0.05.

## Results

### The co-DEGs between tumor samples and normal samples

**Fig. 1:**
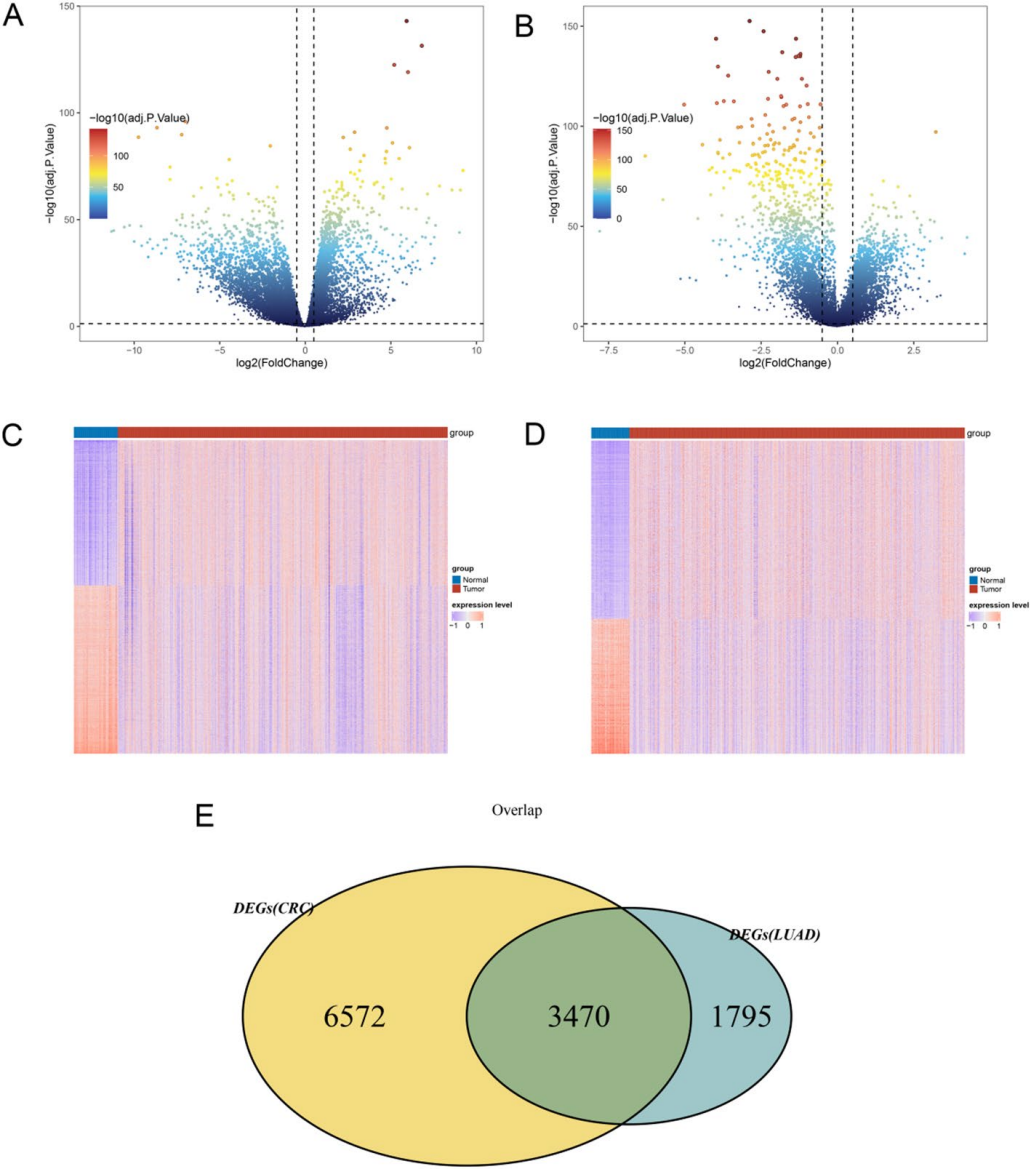
The common-differentially expressed genes (co-DEGs) between tumor samples and normal samples **A**, the volcano plot for DEGs between CRC samples and normal samples. **B**, the volcano plot for DEGs between LUAD samples and normal samples. **C**, the heatmap showed that all these DEGs could be well separated by groups in both CRC dataset. **D**, the heatmap showed that all these DEGs could be well separated by groups in both LUAD dataset

Applying a threshold of *P* < 0.05 and|log2FC| >0.5, a total of 10,042 DEGs were identified in the CRC dataset (Fig. 1A), and 5,265 DEGs were found in the LUAD dataset (Fig. 1B) between tumor and normal samples. The result of heatmap analysis showed that all these DEGs could be well separated by groups in both CRC dataset (Fig. 1C) and LUAD dataset (Fig. 1D). Finally, the VENN plot analysis revealed 3470 co-DEGs based on DEGs from CRC dataset and LUAD dataset (Fig. 1E).

### Enrichment analysis

**Fig. 2:**
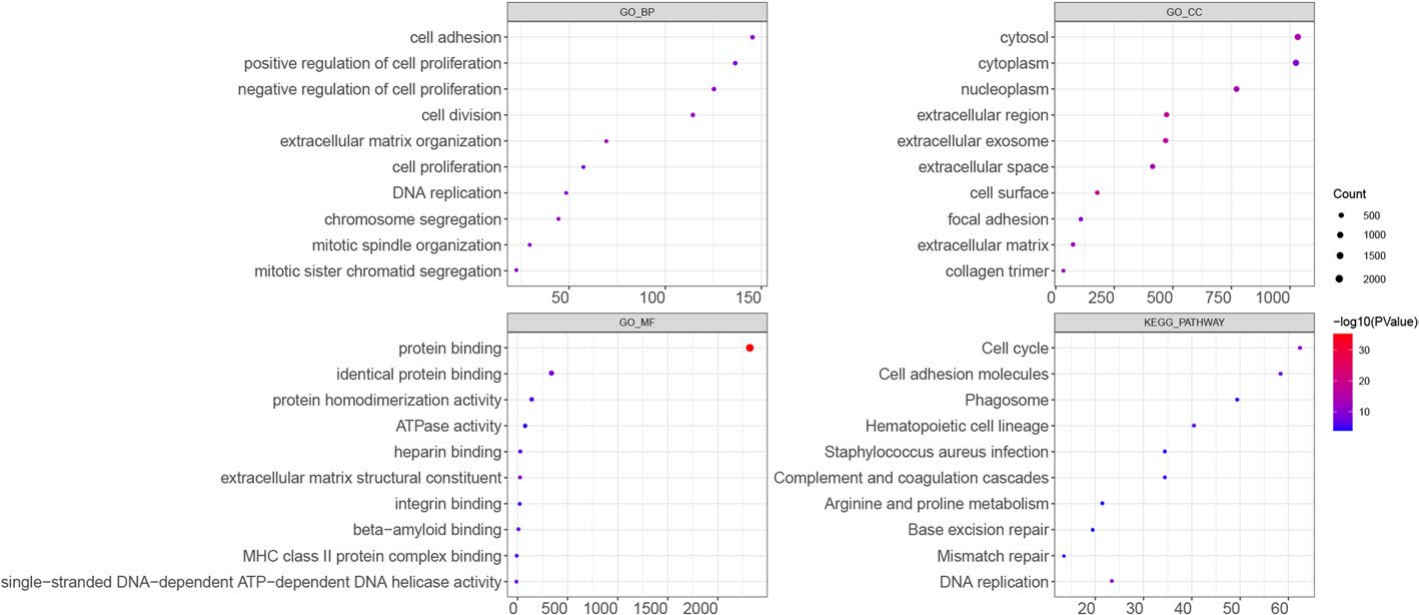
The enrichment analysis on co-DEGs In each chart, the X-axis represented the number of genes assembled in certain item, while the Y-axis represented the name of function or pathway. The larger the node, the more number of gene assembled. The redder the color, the more significant the *P* value

The Top 10 results showed that co-genes were mostly assembled in GO functions like cell adhesion (BP, GO:0007155, Genes: *SPON1*, *SIRPG*, *ICAM5*, etc.), cytosol (CC, GO:0005829, Genes: *NUP107*, *NOC2L*, *MYLK*, etc.) and protein binding (MF, GO:0005515, Genes: *HSPA6*, *SMAD9*, *POP1*, etc.). In addition, these genes were mainly enriched in pathways including cell cycle (hsa04110), cell adhesion molecules (hsa04514) and phagosome (hsa04145) (Fig. 2, Supplemental file 1).

### K-M survival investigation

K-M analysis was performed to evaluate the prognostic value of all co-genes. The result showed that totally 127 and 891 genes were related to patient prognosis in CRC and LUAD, respectively. Finally, the VENN plot analysis revealed totally 28 common prognostic genes (hub genes) that used for subsequent analysis (Supplementary Fig. 1).

### PPI network and functional similarity analysis

**Fig. 3:**
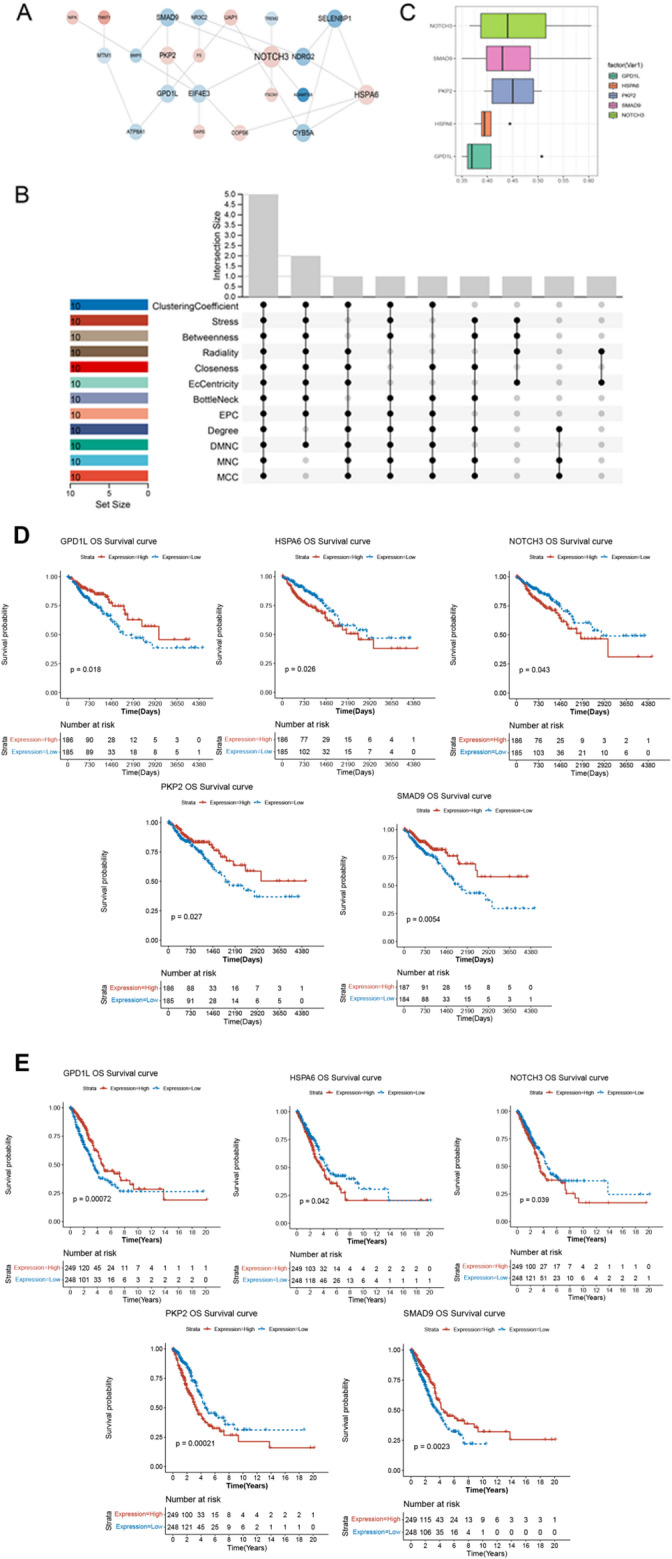
The protein-protein interaction network and functional similarity analysis **A**, the PPI network constructed by the hub genes. The line between two nodes represented the interaction. **B**, topological property analysis revealed five biomarkers. **C**, the functional similarity analysis based on five biomarkers. **D**. Kaplan-Meier (K-M) survival analysis of five biomarkers in CRC. **E**, Kaplan-Meier (K-M) survival analysis of five biomarkers in LUAD. The red line represented high expression of signature gene, while blue line represented low expression of signature gene. The X-axis represented the overall survival (OS) time of patients, while the Y-axis represented the survival probability. *P* < 0.05 was considered as significantly difference

A PPI network was built using 28 hub genes, revealing 24 interactions within the network (Fig. 3A). Then, a topological property analysis was conducted on the PPI network (Fig. 3B). The intersecting investigation for TOP10 genes in each algorithm explored a total of five biomarkers including *HSPA6*, *NOTCH3*, *PKP2*, *SMAD9* and *GPD1L*. Functional similarity analysis revealed that the five biomarkers exhibited a high degree of similarity in their biological roles, with NOTCH3 showing the highest similarity (Fig. 3C). Patients were divided into high and low expression group based on the median of single gene expression of biomarkers. In CRC, the high expressions of GPD1L, PKP2 and SMAD9 and low expression of HSPA6 and NOTCH3 were closely related with better survival rate (all *P* < 0.05, Fig. 3D). In addition, survival curves of LUAD patients showed higher survival probability in high expression group of GPD1L and SMAD9, compared to low expression group. Whereas, the low expression group of HSPA6, NOTCH3 and PKP2 showed a significantly better survival probability compared to high expression groups (all *P* < 0.05, Fig. 3E).

### Expression analysis of biomarkers between tumor samples and normal samples

**Fig. 4:**
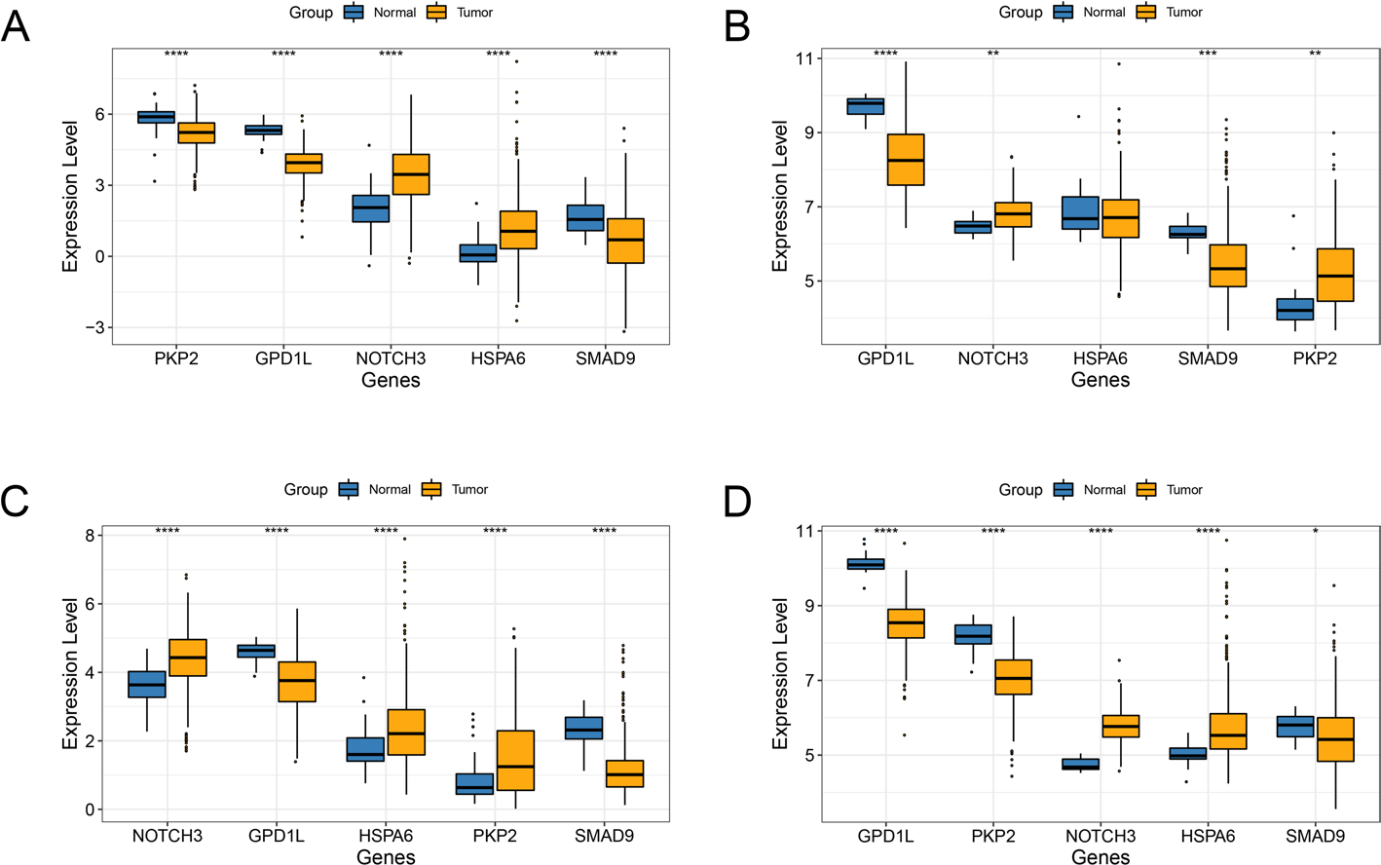
The expression analysis for five biomarkers in CRC/LUAD training dataset and CRC/LUAD validation dataset **A**, the expression of biomarkers between tumor samples and normal samples in CRC training dataset. **B**, the expression of biomarkers between tumor samples and normal samples in CRC validation dataset. **C**, the expression of biomarkers between tumor samples and normal samples in LUAD training dataset. **D**, the expression of biomarkers between tumor samples and normal samples in LUAD validation dataset. *, *P* < 0.05; **, *P* < 0.01; ***, *P* < 0.001; ****, *P* < 0.0001

The expression of five biomarkers between tumor and control samples was investigated in two CRC datasets and two LUAD datasets. The results showed that, compared to normal samples, *HSPA6* and *NOTCH3* were dramatically increased, while *GPD1L*, *PKP2*, and *SMAD9* were dramatically decreased in tumor samples in both the CRC training dataset (Fig. 4A) and the CRC validation dataset (Fig. 4B) (all *P* < 0.05). Moreover, compared to normal samples, *HSPA6*, *NOTCH3*, and *PKP2* were dramatically overexpressed, while *GPD1L* and *SMAD9* were dramatically de-expressed in tumor samples in both the LUAD training dataset (Fig. 4C) and the LUAD validation dataset (Fig. 4D) (all *P* < 0.05).

### Univariate and multivariate analysis of biomarkers

**Fig. 5:**
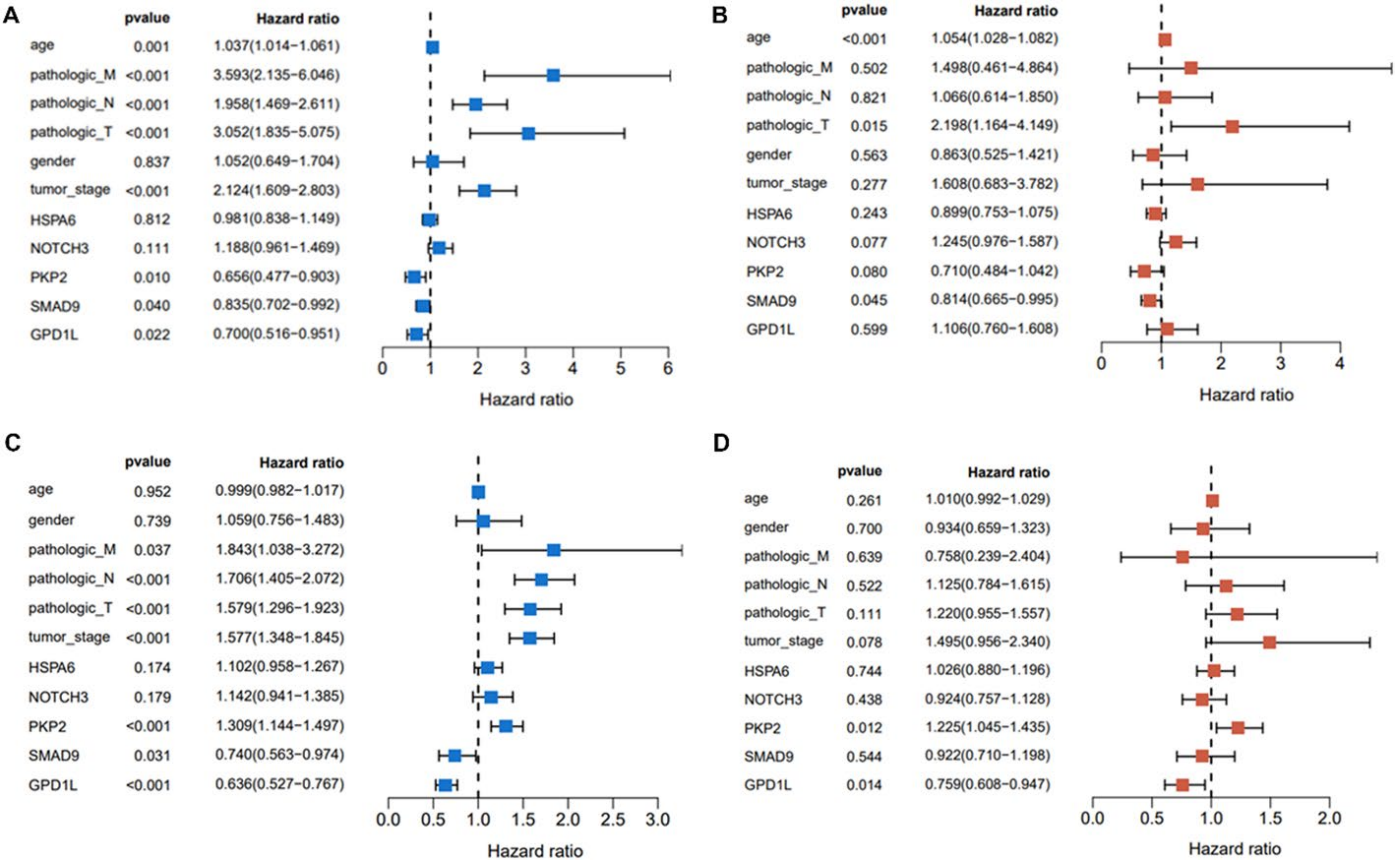
Univariate and multivariate Cox regression analysis of factors associated with survival outcomes. **A**, forest plot of univariate analysis results in CRC; **B**, forest plot of multivariate analysis in CRC. **C**, forest plot of univariate analysis in LUAD; **D**, forest plot of multivariate analysis in LUAD

To explore whether the biomarkers were the independent factors for prognosis, univariate and multivariate analyses were performed combined with clinical variables. As illustrated in Fig. 5A, higher pathologic T/N/M and increased tumor stage were closely associated with poor prognosis of CRC. Lower expressions of PKP2, SMAD9 and GPD1L were associated with improved survival (all *p* < 0.05). In multivariate Cox regression adjusted for age, gender, pathologic T/N/M and tumor stage, SMAD9 remained the independent risk factor for the outcomes of CRC (Fig. 5B). For LUAD, univariate and multivariate analyses demonstrated that PKP2 and GPD1L were the independent prognostic factors (all *p* < 0.05, Fig. 5C and D).

### Immune infiltration analysis based on biomarkers

**Fig. 6:**
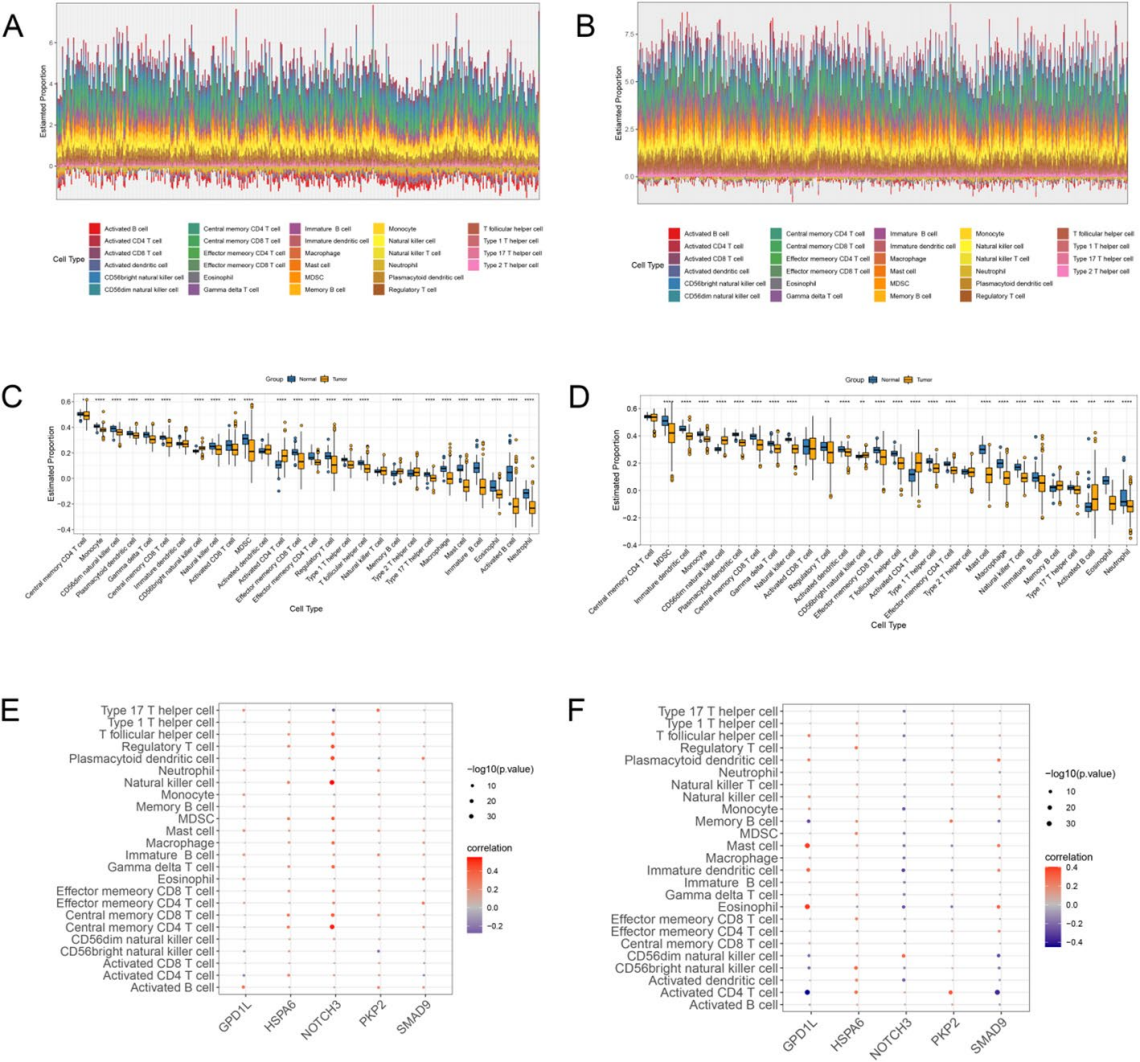
Immunological analysis based on biomarkers **A**, the immune cell infiltration abundance analysis of 28 immune cells in CRC dataset based on ssGSEA algorithm. **B**, the immune cell infiltration abundance analysis of 28 immune cells in LUAD dataset based on ssGSEA algorithm. **C**, the box plot showed 24 immune cells that differentially expressed between tumor samples and normal samples in CRC dataset. **D**, the box plot showed 25 immune cells that differentially expressed between tumor samples and normal samples in LUAD dataset. **E**, the bubble plot showed the correlation between immune cells and biomarkers. The bigger the node, the more significant the P value. The redder the color, the higher the correlation. *, *P* < 0.05; **, *P* < 0.01; ***, *P* < 0.001; ****, *P* < 0.0001

Immune cell infiltration abundance for all 28 immune cells in CRC training dataset and LUAD training dataset was showed in Fig. 6A and B, respectively. The results obtained based on ssGSEA analysis revealed that totally 24 kinds of immune cells (Fig. 6C) and 25 kinds of immune cells (Fig. 6D) were significant differentially expressed between two samples in CRC dataset and LUAD dataset, respectively (all *P* < 0.05). For example, compared to the control group, activated CD4 + T cells were dramatically up-regulated in both CRC tumor samples and LUAD tumor samples (all *P* < 0.0001). However, compared to normal samples, Mast cells were significantly down-regulated in both CRC tumor samples and LUAD tumor samples (all *P* < 0.0001). These findings indicated distinct immune cell profiles associated with CRC and LUAD patients. Then, the correlation between biomarker and immune cell based on CRC dataset and LUAD dataset. According to the ranking of correlation coefficients, the results indicated a strong positive correlation between *NOTCH3* and natural killer cells in the CRC dataset (Fig. 6E). In addition, in LUAD dataset, there was a there was a strongest significant positive correlation between *GPD1L* and Eosinophil (Fig. 6F).

### GSEA and target drug analysis based on biomarkers

**Fig. 7:**
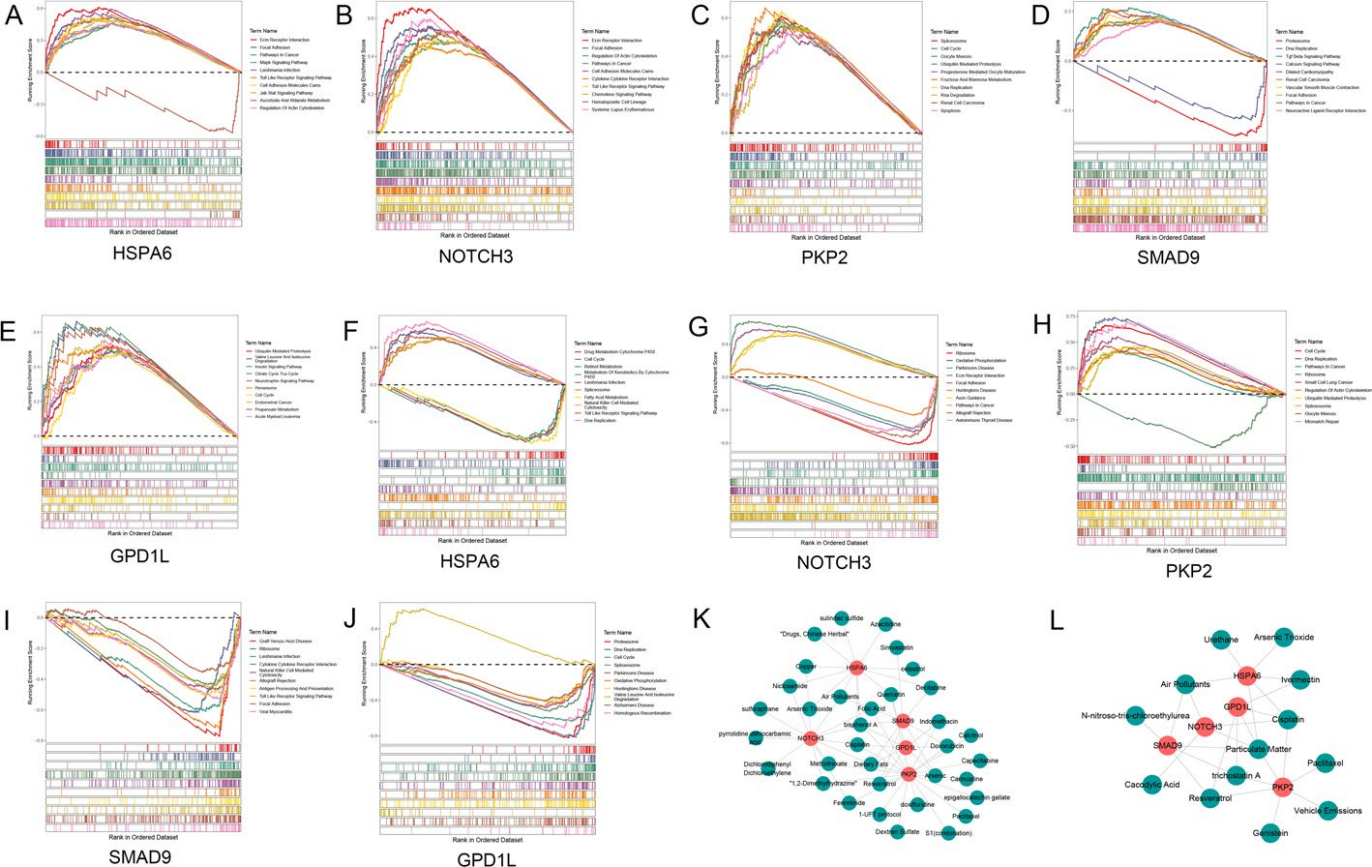
GSEA and target drug analysis based on biomarkers **A-E**, the TOP10 result of enrichment for five biomarkers including HSPA6, NOTCH3, PKP2, SMAD9 and GPD1L in CRC training dataset, respectively. **F-J**, the TOP10 result of enrichment for five biomarkers including HSPA6, NOTCH3, PKP2, SMAD9 and GPD1L in LUAD training dataset, respectively. The X-axis represented the ranking of gene fold changes, with each small vertical line representing a gene. **K**, the gene-drug interaction network constructed by five biomarkers and CRC-associated drugs. **L**, the gene-drug interaction network constructed by five biomarkers and LUAD-associated drugs. The red node presented biomarker, while the green node represented drug

The GSEA enrichment analysis was performed based CRC training dataset and LUAD training dataset. With *P* < 0.05, the TOP10 result of enrichment for *HSPA6*, *NOTCH3*, *PKP2*, *SMAD9* and *GPD1L* in CRC training dataset were showed in Fig. 7A-E, respectively. In addition, the TOP10 result of enrichment for all five biomarkers in LUAD training dataset were showed in Fig. 7F-G. Furthermore, the gene-drug interactions were predicated based on all five biomarkers and CTD database. Finally, the result showed that there were 34 CRC drugs (Fig. 7K) and 13 LUAD drugs (Fig. 7L) were associated with five biomarkers in current analysis.

### Mice model construction

After mice model construction for 3 weeks, the DAI score of CRC mice was calculated. DAI score was significantly increased in CRC group, compared with control group (*p* < 0.001, Supplementary Fig. 2A). The tumor volume of LUAD mice was markedly increased, compared to control group (*p* < 0.001, Supplementary Fig. 2B). In the experimental process, two CRC mice with DAI score = 4 were dead and 3 LUAD mice died for respiratory distress or excessive weight loss (Supplementary Fig. 2C and D). To maintain *n* = 5/group, the dead mice were replaced with new cohorts under identical conditions.

### The qRT-PCR analysis

**Fig. 8:**
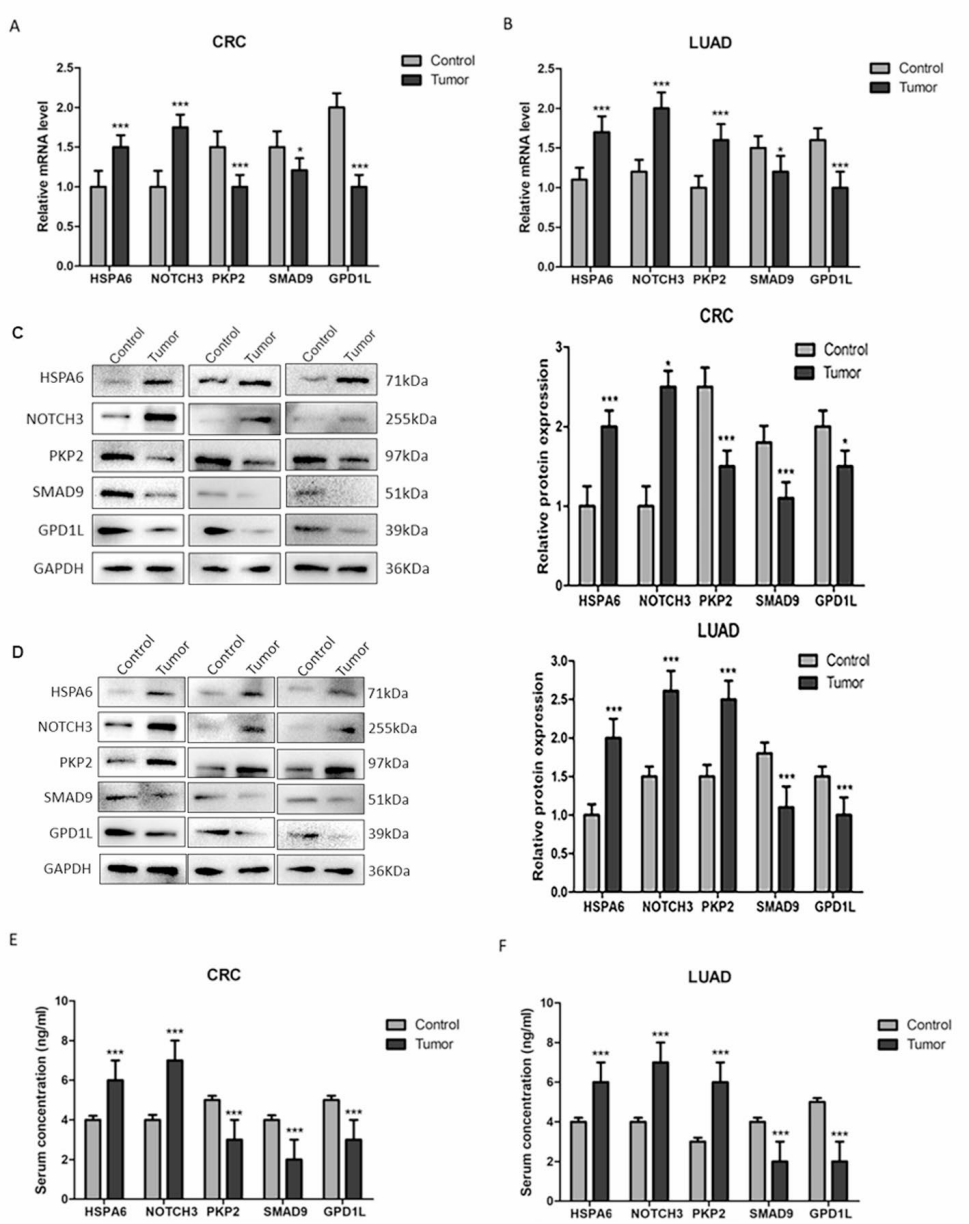
The validation analysis for five biomarkers based on cell lines and mice model **A**, the qRT-PCR analysis for the relative mReRNA expression of five biomarkers between tumor samples and control samples in CRC. **B**, the qRT-PCR analysis for the relative mRNA expression of five biomarkers between tumor samples and control samples in LUAD. **C**, the Western blot analysis for the protein expression of five biomarkers between tumor samples and control samples in CRC. **D**, the Western blot analysis for the protein expression of five biomarkers between tumor samples and control samples in LUAD. **E**, the ELISA analysis showed the serum concentration for biomarkers between tumor samples and control samples in CRC. **F**, the ELISA analysis showed the serum concentration for biomarkers between tumor samples and control samples in LUAD. *, *P* < 0.05 when compared with control group. ***, *P* < 0.01 when compared with control group

The result demonstrated a significant increase in HSPA6 and NOTCH3 expression, but a significant decreased in *GPD1L*, *PKP2* and *SMAD9* in CRC tumor group when compared to those in normal group (all *P* < 0.001) (Fig. 8A). Meanwhile, when compared with normal group, the *HSPA6*, *NOTCH3* and *PKP2* expression were significantly increased in LUAD tumor group, but *GPD1L* and *SMAD9* expression were dramatically decreased in control group (all *P* < 0.001) (Fig. 8B). The results of verification analysis were consistent with the findings of our current bioinformatic study, affirming the reliability of our results.

### Western blotting investigation

In the CRC tumor group, the expression levels of *HSPA6* and *NOTCH3* protein were dramatically up-regulated, while *GPD1L*, *PKP2*, and *SMAD9* protein were dramatically down-regulated (all *P* < 0.05) (Fig. 8C, Supplementary file 2). Similarly, in the LUAD tumor group, *HSPA6*, *NOTCH3*, and *PKP2* protein expression were dramatically stimulated, whereas *GPD1L* and *SMAD9* protein expression were dramatically suppressed (all *P* < 0.05) (Fig. 8D, Supplementary file 3).

### ELISA investigation

To assess the clinical application potential of these biomarkers, ELISA was employed to measure the protein levels encoded by the biomarkers in animal samples. Compared to the control group, serum levels of *HSPA6* and *NOTCH3* were significantly elevated in the CRC tumor group, whereas *GPD1L*, *PKP2*, and *SMAD9* exhibited a marked decrease (Fig. 8E). In addition, the expression of serum HSPA6, *NOTCH3* and *PKP2 were* significantly up-regulated in the LUAD tumor group, while *GPD1L* and *SMAD9* showed a significant down-regulation trend when compared to the control group (Fig. 8F). These findings indicated that all five biomarkers had diagnostic value for CRC and LUAD.

## Discussion

CRC and LUAD are among the most serious global health threats. Although the significant genetic link between two disease has been revealed [4], there remains a limited exploration of cross-cancer signatures and associated molecular mechanism on CRC and LUAD clinical diagnosis/therapy. In this study, we explored totally 3470 co-DEGs of tumor vs. normal based on CRC and LUAD dataset. K-M survival and PPI network analysis revealed five hub prognostic genes, including *HSPA6*, *NOTCH3*, *PKP2*, *SMAD9*, and *GPD1*, as biomarkers for two diseases, with enrichment analysis showing that biomarkers like *HSPA6* and *SMAD9* were primarily associated with protein binding functions. Functional similarity analysis revealed that the five biomarkers exhibited a high degree of similarity in their biological roles, with *NOTCH3* showing the highest similarity, further supported by immune infiltration analysis indicating a significant and strong positive correlation between *NOTCH3* and Natural Killer cells. Finally, the expression investigation of five biomarkers based on cell lines and mice model validated the results of bioinformatics analysis.

Heat shock protein family A (HSP70) member 6 (*HSPA6*) plays a central role in the cellular response to stress [29]. A previous study shows that the excessive HSP70 activation leads to remodeling of the TME to resist chemotherapy sensitivity in CRC [30]. *HSPA6* has been identified as a crucial regulator of cell sensitivity, likely through its interactions with other members of the HSPA family during lung cancer progression [31]. A recent evidence determined that the upregulation of HSPA6 was associated with the malignant progression of NSCLC [32]. Our data suggested that HSPA6 was upregulated in LUAD compared to controls and its high expression indicated poor prognosis by survival curve analysis. Our results were consistent with the previous report. Plakophilin 2 (*PKP2*) is a desmosomal protein that mainly participates in the maintenance of intercellular junctions and the preservation of structural integrity [33]. Hao et al. indicated that the LUAD progression was promoted by the up-regulation of *PKP2* [34]. The high expression of PKP2 was closely associated with unfavorable outcome in LUAD patients [35], which was in align with our survival analysis. A recent study proves that downregulation of *PKP2* is significantly associated with adverse immune infiltration and poorer survival outcomes in colon adenocarcinoma [36]. The multiple roles of *PKP2* in maintaining cell structural stability and signal transduction make it a potential diagnostic and prognostic biomarker. Mothers against decapentaplegic homolog 9 (*SMAD9*) is extensively contribute to the cell differentiation and proliferation [37]. It has been proved that SMAD9 knockdown successfully abrogated the promotion of Chondroitin polymerizing factor overexpression in CRC progression, indicating an important role of *SMAD9* in CRC [38]. The low-expression of *SMAD9* was significantly associated with the low survival of patients with LUAD [39], which was in agreement with our survival analysis. Glycerol-3-phosphate dehydrogenase 1-like protein (*GPD1L*) is a gene mainly involved in cellular energy metabolism and redox reactions. Fan et al. indicated that the stimulation of *GPD1L* significantly inhibited the LUAD cells’ proliferation, which was further demonstrated by qRT-PCR analysis, suggesting that *GPD1L* could be a potential prognostic biomarker for LUAD [40]. The prognostic value of *GPD1L* has also been proved in CRC based on a previous bioinformatics analysis based on TCGA database [41]. In addition, Notch receptor 3 (*NOTCH3*) influences the survival of tumor cells in various cancers. A recent single-cell investigation shows that *NOTCH3* contribute to the microenvironment remodeling and invasion in LUAD via mediating the interactions between stromal cells [42]. It has been proved that *NOTCH3* signaling promotes the tumor growth and closely associated with the poor outcome in patients with CRC [43]. Actually, as a prognostic biomarker, *NOTCH3* may contribute to tumor progression via participating in immune infiltration process in human cancer [44].

In this study, the biomarkers were strongly associated with immune cells in immune infiltration studies and held significant prognostic value. Validation analyses in cellular and animal models confirmed the bioinformatics findings for these five biomarkers. Therefore, we hypothesize that these biomarkers may play crucial roles in the development and progression of CRC and LUAD through various mechanisms, including influencing cellular stress responses, signal transduction, and TME, thereby providing new directions for future cancer research and clinical applications.

Our GO enrichment results showed that *HSPA6* and *SMAD9* were primarily associated with protein binding, suggesting that these genes may play critical roles in the molecular mechanisms underlying both CRC and LUAD. The protein binding function is essential for various cellular processes, including signal transduction, molecular chaperoning, and the regulation of transcriptional activity [45]. *HSPA6*, a member of the heat shock protein family, is known for its role in protecting cells from stress by assisting in the proper folding of proteins and preventing protein aggregation [46]. This chaperone activity could be crucial in the tumor microenvironment, where cellular stress is prevalent [47]. On the other hand, *SMAD9*, a key mediator in the TGF-β/BMP signaling pathway, regulates gene expression through interactions with other proteins, influencing cell proliferation and differentiation [48]. The involvement of *SMAD9* in protein binding functions may underscore its role in modulating signaling pathways that are often dysregulated in cancer. Therefore, the association of *HSPA6* and *SMAD9* with protein binding functions highlights their potential as key players in the pathogenesis of CRC and LUAD, providing insights into common molecular mechanisms that could be targeted for therapeutic intervention. Furthermore, our functional similarity analysis revealed that the five biomarkers studied exhibited a high degree of similarity in their biological roles, with *NOTCH3* demonstrating the highest similarity. This finding aligns with existing research, which suggests that *NOTCH3* is integral to various oncogenic processes, including cell fate determination, proliferation, and survival, particularly in the context of LUAD [49]. The convergence of functional roles among these biomarkers, particularly in protein binding, underscores a potentially shared pathway in CRC and LUAD pathogenesis, which further shared mechanism that could open new pathways for therapeutic targeting. Notably, by univariate and multivariate regression analysis, SMAD9 remained an independent prognostic factor in CRC after adjusting for pathologic stages, suggesting its potential role beyond conventional clinicopathological parameters. Similarly, PKP2 and GPD1L retained significance in LUAD, supporting their utility as biomarkers even when accounting for tumor progression. However, there were some limitations in the current study. First, there is a lack of long-term follow-up clinical data to further confirm the actual prognostic value of these biomarkers, as well as the inability to comprehensively cover all possible co-biomarkers and their related mechanisms due to limitations in time and resources. Therefore, a further validation with a larger sample size is necessary. Secondly, although our multivariate Cox regression adjusted for key clinical variables (e.g., age, gender, pathologic T/N/M stage, and tumor stage), other unmeasured confounders, such as treatment regimens, comorbidities, or molecular heterogeneity, might influence the prognostic value of PKP2, SMAD9, and GPD1L. Future studies incorporating more comprehensive clinical and molecular data are warranted to validate these findings. Finally, while the five biomarker proteins (HSPA6, NOTCH3, PKP2, SMAD9, and GPD1L) are primarily intracellular, their presence in serum could potentially be attributed to tumor derived extracellular vesicles or cell lysis during tumor progression. Currently, the evidences of the presence of these proteins are insufficient. Due to current experimental constraints, the presence of these proteins in serum is not confirmed in our study, which may limit the clinical applications of the biomarkers. In the future, we would like to perform additional validation experiments to definitively confirm the serum level of these proteins.

In conclusion, *HSPA6*, *NOTCH3*, *PKP2*, *SMAD9*, and *GPD1* were five novel biomarkers for CRC and LUAD clinical diagnosis or treatment. Moreover, *HSPA6* and *SMAD9* might take part in the progression of CRC and LUAD via protein binding function.

## Electronic supplementary material

Supplementary Material 1.

Supplementary Material 2.

Supplementary Material 3.

## Data Availability

The original data is available from the corresponding author upon reasonable request.

## References

[1] Shafieipour S, Zamanian Y, Hadipour E, Sinaei R, Khoshnazar SM. Exploring the effects of alpha-pinene on apoptosis induction in human colon cancer cells via the PI3K/AKT signaling pathway: an in vitro study. Egypt J Med Hum Genet. 2025;26(1):27.

[2] Wang R, Dai W, Gong J, Huang M, Hu T, Li H, et al. Development of a novel combined nomogram model integrating deep learning-pathomics, radiomics and immunoscore to predict postoperative outcome of colorectal cancer lung metastasis patients. J Hematol Oncol. 2022;15(1):11. 10.1186/s13045-022-01225-3. 35073937 10.1186/s13045-022-01225-3 PMC8785554

[3] Dragani TA, Muley T, Schneider MA, Kobinger S, Eichhorn M, Winter H, et al. Lung adenocarcinoma diagnosed at a younger age is associated with advanced stage, female sex, and Ever-Smoker status, in patients treated with lung resection. Cancers. 2023;15(8). 10.3390/cancers15082395. 10.3390/cancers15082395 PMC10136510 37190323

[4] Xiao Z, Wang Z, Zhang T, Liu Y, Si M. Bidirectional Mendelian randomization analysis of the genetic association between primary lung cancer and colorectal cancer. J Translational Med. 2023;21(1):722. 10.1186/s12967-023-04612-7. 10.1186/s12967-023-04612-7 PMC10577972 37840123

[5] Bao Z, Zeng W, Zhang D, Wang L, Deng X, Lai J, et al. SNAIL induces EMT and lung metastasis of tumours secreting CXCL2 to promote the invasion of M2-Type immunosuppressed macrophages in colorectal Cancer. Int J Biol Sci. 2022;18(7):2867–81. 10.7150/ijbs.66854. 35541899 10.7150/ijbs.66854 PMC9066124

[6] Constâncio V, Nunes SP, Henrique R, Jerónimo C. DNA Methylation-Based testing in liquid biopsies as detection and prognostic biomarkers for the four major Cancer types. Cells. 2020;9(3). 10.3390/cells9030624. 10.3390/cells9030624 PMC7140532 32150897

[7] Giopanou I, Pintzas A. RAS and BRAF in the foreground for non-small cell lung cancer and colorectal cancer: similarities and main differences for prognosis and therapies. Crit Rev Oncol/Hematol. 2020;146. 10.1016/j.critrevonc.2019.102859. 10.1016/j.critrevonc.2019.102859 31927392

[8] Oura K, Morishita A, Tani J, Masaki T. Tumor immune microenvironment and immunosuppressive therapy in hepatocellular carcinoma: A review. Int J Mol Sci. 2021;22(11). 10.3390/ijms22115801. 10.3390/ijms22115801 PMC8198390 34071550

[9] Dai W, Guo C, Wang Y, Li Y, Xie R, Wu J, et al. Identification of hub genes and pathways in lung metastatic colorectal cancer. BMC Cancer. 2023;23(1):323. 10.1186/s12885-023-10792-8. 37024866 10.1186/s12885-023-10792-8 PMC10080892

[10] Larson NB, Oberg AL, Adjei AA, Wang L. A clinician’s guide to bioinformatics for Next-Generation sequencing. J Thorac Oncology: Official Publication Int Association Study Lung Cancer. 2023;18(2):143–57. 10.1016/j.jtho.2022.11.006. 10.1016/j.jtho.2022.11.006 PMC9870988 36379355

[11] Mortezapour M, Tapak L, Bahreini F, Najafi R, Afshar S. Identification of key genes in colorectal cancer diagnosis by co-expression analysis weighted gene co-expression network analysis. Comput Biol Med. 2023;157:106779. 10.1016/j.compbiomed.2023.106779. 36931200 10.1016/j.compbiomed.2023.106779

[12] Lee SC, Quinn A, Nguyen T, Venkatesh S, Quinn TP. A cross-cancer metastasis signature in the microRNA-mRNA axis of paired tissue samples. Mol Biol Rep. 2019;46(6):5919–30. 10.1007/s11033-019-05025-w. 31410687 10.1007/s11033-019-05025-w

[13] Wang Z, Jensen MA, Zenklusen JC. A practical guide to the Cancer genome atlas (TCGA). Methods in molecular biology. (Clifton NJ). 2016;1418:111–41. 10.1007/978-1-4939-3578-9_6. 10.1007/978-1-4939-3578-9_6 27008012

[14] Ritchie ME, Phipson B, Wu D, Hu Y, Law CW, Shi W, et al. Limma powers differential expression analyses for RNA-sequencing and microarray studies. Nucleic Acids Res. 2015;43(7):e47. 10.1093/nar/gkv007. 25605792 10.1093/nar/gkv007 PMC4402510

[15] Ito K, Murphy D. Application of ggplot2 to pharmacometric graphics. CPT: Pharmacometrics Syst Pharmacol. 2013;2(10):e79. 10.1038/psp.2013.56. 24132163 10.1038/psp.2013.56 PMC3817376

[16] Chen H, Boutros PC. VennDiagram: a package for the generation of highly-customizable Venn and Euler diagrams in R. BMC Bioinformatics. 2011;12:35. 10.1186/1471-2105-12-35. 21269502 10.1186/1471-2105-12-35 PMC3041657

[17] Chen L, Zhang YH, Wang S, Zhang Y, Huang T, Cai YD. Prediction and analysis of essential genes using the enrichments of gene ontology and KEGG pathways. 2017;12(9):e018412910.1371/journal.pone.0184129 10.1371/journal.pone.0184129 PMC5584762 28873455

[18] Sherman BT, Hao M, Qiu J, Jiao X, Baseler MW, Lane HC, et al. DAVID: a web server for functional enrichment analysis and functional annotation of gene lists (2021 update). Nucleic Acids Res. 2022;50(W1):W216–21. 10.1093/nar/gkac194. 35325185 10.1093/nar/gkac194 PMC9252805

[19] Bland JM, Altman DG. Survival probabilities (the Kaplan-Meier method). BMJ. 1998;317(7172):1572. 9836663 10.1136/bmj.317.7172.1572 PMC1114388

[20] Szklarczyk D, Franceschini A, Wyder S, Forslund K, Heller D, Huerta-Cepas J et al. STRING v10: protein–protein interaction networks, integrated over the tree of life. Nucleic Acids Res. 2015;43(0):D447-52. 10.1093/nar/gku1003 PMC4383874 25352553

[21] Chin CH, Chen SH, Wu HH, Ho CW, Ko MT, Lin CY. CytoHubba: identifying hub objects and sub-networks from complex interactome. BMC Syst Biol. 2014;8(Suppl 4):S11. 25521941 10.1186/1752-0509-8-S4-S11 PMC4290687

[22] Hänzelmann S, Castelo R, Guinney J. GSVA: gene set variation analysis for microarray and RNA-seq data. BMC Bioinformatics. 2013;14:7. 10.1186/1471-2105-14-7. 23323831 10.1186/1471-2105-14-7 PMC3618321

[23] Pripp AH. [Pearson’s or Spearman’s correlation coefficients]. Tidsskr nor Laegeforen. 2018;138(8). 10.4045/tidsskr.18.0042. 10.4045/tidsskr.18.0042 29737766

[24] Liberzon A, Subramanian A, Pinchback R, Thorvaldsdóttir H, Tamayo P, Mesirov JP. Molecular signatures database (MSigDB) 3.0. Bioinformatics. 2011;27(12):1739–40. 21546393 10.1093/bioinformatics/btr260 PMC3106198

[25] Davis AP, Wiegers TC, Johnson RJ, Sciaky D, Wiegers J, Mattingly CJ. Comparative toxicogenomics database (CTD): update 2023. Nucleic Acids Res. 2023;51(D1):D1257. 10.1093/nar/gkac833. 36169237 10.1093/nar/gkac833 PMC9825590

[26] Mattingly CJ, Colby GT, Forrest JN, Boyer JL. The comparative toxicogenomics database (CTD). Environ Health Perspect. 2003;111(6):793–5. 12760826 10.1289/ehp.6028 PMC1241500

[27] Cheng J, Zhang L, Dai W, Mao Y, Li S, Wang J, et al. Ghrelin ameliorates intestinal barrier dysfunction in experimental colitis by inhibiting the activation of nuclear factor-kappa B. Biochem Biophys Res Commun. 2015;458(1):140–7. 25634696 10.1016/j.bbrc.2015.01.083

[28] Cooper HS, Murthy SN, Shah RS, Sedergran DJ. Clinicopathologic study of dextran sulfate sodium experimental murine colitis. Lab Invest. 1993;69(2):238–49. 8350599

[29] Song B, Shen S, Fu S, Fu J. HSPA6 and its role in cancers and other diseases. Mol Biol Rep. 2022;49(11):10565–77. 10.1007/s11033-022-07641-5. 35666422 10.1007/s11033-022-07641-5

[30] Feng H, Guo Z, Chen X, Liu K, Li H, Jia W et al. Excessive HSP70/TLR2 activation leads to remodeling of the tumor immune microenvironment to resist chemotherapy sensitivity of mFOLFOX in colorectal cancer. Clinical immunology (Orlando, Fla). 2022;245:10915710.1016/j.clim.2022.109157 10.1016/j.clim.2022.109157 36244673

[31] Wang L, Hou J, Wang J, Zhu Z, Zhang W, Zhang X, et al. Regulatory roles of HSPA6 in Actinidia chinensis planch. Root extract (acRoots)-inhibited lung cancer proliferation. Clin Translational Med. 2020;10(2):e46. 10.1002/ctm2.46. 10.1002/ctm2.46 PMC7403824 32508044

[32] Zhu X, Chen X, Shen X, Liu Y, Fu W, Wang B, et al. PP4R1 accelerates the malignant progression of NSCLC via up-regulating HSPA6 expression and HSPA6-mediated ER stress. Biochimica et biophysica acta (BBA)-Molecular. Cell Res. 2024;1871(1):119588. 10.1016/j.bbamcr.2023.119588 37739270

[33] Nagler S, Ghoreishi Y, Kollmann C, Kelm M, Gerull B, Waschke J, et al. Plakophilin 2 regulates intestinal barrier function by modulating protein kinase C activity in vitro. Tissue Barriers. 2023;11(4):2138061. 10.1080/21688370.2022.2138061. 36280901 10.1080/21688370.2022.2138061 PMC10606776

[34] Hao XL, Tian Z, Han F, Chen JP, Gao LY, Liu JY. Plakophilin-2 accelerates cell proliferation and migration through activating EGFR signaling in lung adenocarcinoma. Pathol Res Pract. 2019;215(7):152438. 10.1016/j.prp.2019.152438. 31126818 10.1016/j.prp.2019.152438

[35] Wu Y, Liu L, Shen X, Liu W, Ma R. Plakophilin-2 promotes lung adenocarcinoma development via enhancing focal adhesion and epithelial–mesenchymal transition. Cancer Manage Res. 2021: 13(0):559–70. 10.2147/CMAR.S281663 PMC7837596 33519235

[36] Dai M, Su Y, Wu Z. Downregulated expression of plakophilin-2 gene in patients with colon adenocarcinoma predicts an unfavorable prognosis and immune infiltrate. J Gene Med. 2024;26(1):e3592. 10.1002/jgm.3592. 37726168 10.1002/jgm.3592

[37] Yu D, Zhang L, Wang H, Chen F, Chen J, Zhang Z, et al. A potential role for SMAD9 in Goose follicular selection through regulation of mRNA levels of luteinizing hormone receptor. Theriogenology. 2019;135:204–12. 10.1016/j.theriogenology.2018.11.022. 30522699 10.1016/j.theriogenology.2018.11.022

[38] Zhao J, Tang X, Zhu H. Chondroitin polymerizing factor (CHPF) promotes the progression of colorectal cancer through ASB2-mediated ubiquitylation of SMAD9. Histol Histopathol. 2024. 10.14670/hh-18-738. 38591191 10.14670/HH-18-738

[39] Zhai Y, Zhao B, Wang Y, Li L, Li J, Li X, et al. Construction of the optimization prognostic model based on differentially expressed immune genes of lung adenocarcinoma. BMC Cancer. 2021;21(1):213. 10.1186/s12885-021-07911-8. 33648465 10.1186/s12885-021-07911-8 PMC7923649

[40] Fan Z, Wu S, Sang H, Li Q, Cheng S, Zhu H. Identification of GPD1L as a potential prognosis biomarker and associated with immune infiltrates in lung adenocarcinoma. Mediat Inflamm. 2023;2023:9162249. 10.1155/2023/9162249. 10.1155/2023/9162249 PMC10079383 37035759

[41] Zhao Z, Cui X, Guan G, Liu Y, Liu X, Chen Z, et al. Bioinformatics analysis reveals the clinical significance of GIPC2/GPD1L for colorectal cancer using TCGA database. Translational cancer Res. 2022;11(4):761–71. 10.21037/tcr-21-1933. 10.21037/tcr-21-1933 PMC9091031 35571634

[42] Xiang H, Pan Y, Sze MA, Wlodarska M, Li L, van de Mark KA, et al. Single-Cell analysis identifies NOTCH3-Mediated interactions between stromal cells that promote microenvironment remodeling and invasion in lung adenocarcinoma. Cancer Res. 2024;84(9):1410–25. 10.1158/0008-5472.Can-23-1183. 38335304 10.1158/0008-5472.CAN-23-1183 PMC11063690

[43] Serafin V, Persano L, Moserle L, Esposito G, Ghisi M, Curtarello M, et al. Notch3 signalling promotes tumour growth in colorectal cancer. J Pathol. 2011;224(4):448–60. 10.1002/path.2895. 21598247 10.1002/path.2895

[44] Xu J, Jin XL, Shen H, Chen XW, Chen J, Huang H, et al. NOTCH3 as a prognostic biomarker and its correlation with immune infiltration in Gastrointestinal cancers. Sci Rep. 2024;14(1):14327. 10.1038/s41598-024-65036-x. 38906903 10.1038/s41598-024-65036-x PMC11192884

[45] Qin S, Zhou HX. Protein folding, binding, and droplet formation in cell-like conditions. Curr Opin Struct Biol. 2017;43:28–37. 10.1016/j.sbi.2016.10.006. 27771543 10.1016/j.sbi.2016.10.006 PMC5397379

[46] Shen S, Wei C, Fu J. RNA-Sequencing reveals heat shock 70-kDa protein 6 (HSPA6) as a novel Thymoquinone-Upregulated gene that inhibits growth, migration, and invasion of Triple-Negative breast Cancer cells. Front Oncol. 2021;11:667995. 10.3389/fonc.2021.667995. 34017687 10.3389/fonc.2021.667995 PMC8129564

[47] Zhu X, Chen X, Shen X, Liu Y, Fu W, Wang B, et al. PP4R1 accelerates the malignant progression of NSCLC via up-regulating HSPA6 expression and HSPA6-mediated ER stress. Biochim Et Biophys Acta Mol Cell Res. 2024;1871(1):119588. 10.1016/j.bbamcr.2023.119588. 10.1016/j.bbamcr.2023.119588 37739270

[48] Gong Y, Xu F, Zhang L, Qian Y, Chen J, Huang H, et al. MicroRNA expression signature for Satb2-induced osteogenic differentiation in bone marrow stromal cells. Mol Cell Biochem. 2014;387(1–2):227–39. 10.1007/s11010-013-1888-z. 24218084 10.1007/s11010-013-1888-z

[49] Li Y, Gao Y, Niu X, Tang M, Li J, Song B, et al. LncRNA BASP1-AS1 interacts with YBX1 to regulate Notch transcription and drives the malignancy of melanoma. Cancer Sci. 2021;112(11):4526–42. 10.1111/cas.15140. 34533860 10.1111/cas.15140 PMC8586662

