# Supplementary material for: Investigation of biomarkers and associated molecular mechanism shared between colorectal cancer and lung adenocarcinoma
Source: Discov Oncol. 2025 Aug 12;16:1540. doi: 10.1007/s12672-025-03240-5 (PMC12343392; doi:10.1007/s12672-025-03240-5)
Supplement: Supplementary file 1 — Supplementary Material 1. [file 12672_2025_3240_MOESM1_ESM.docx]

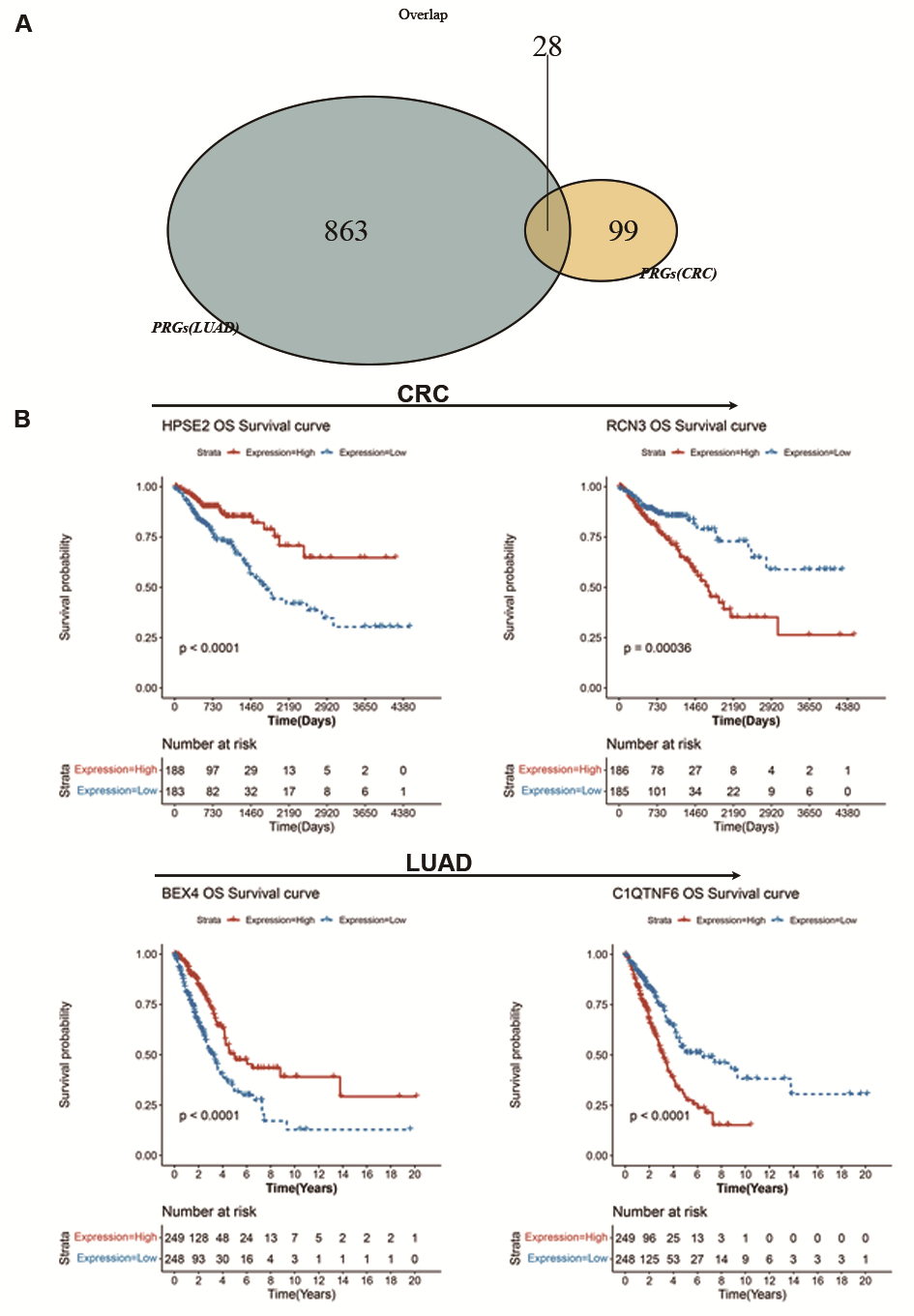


**Supplementary Fig. 1 The 28 common prognostic genes (hub genes) revealed by using VENN plot analysis**

A, Venn diagram image. B, the Top 2 genes closely associated with survival rate (ranked by least P value) in colorectal cancer and lung adenocarcinoma.


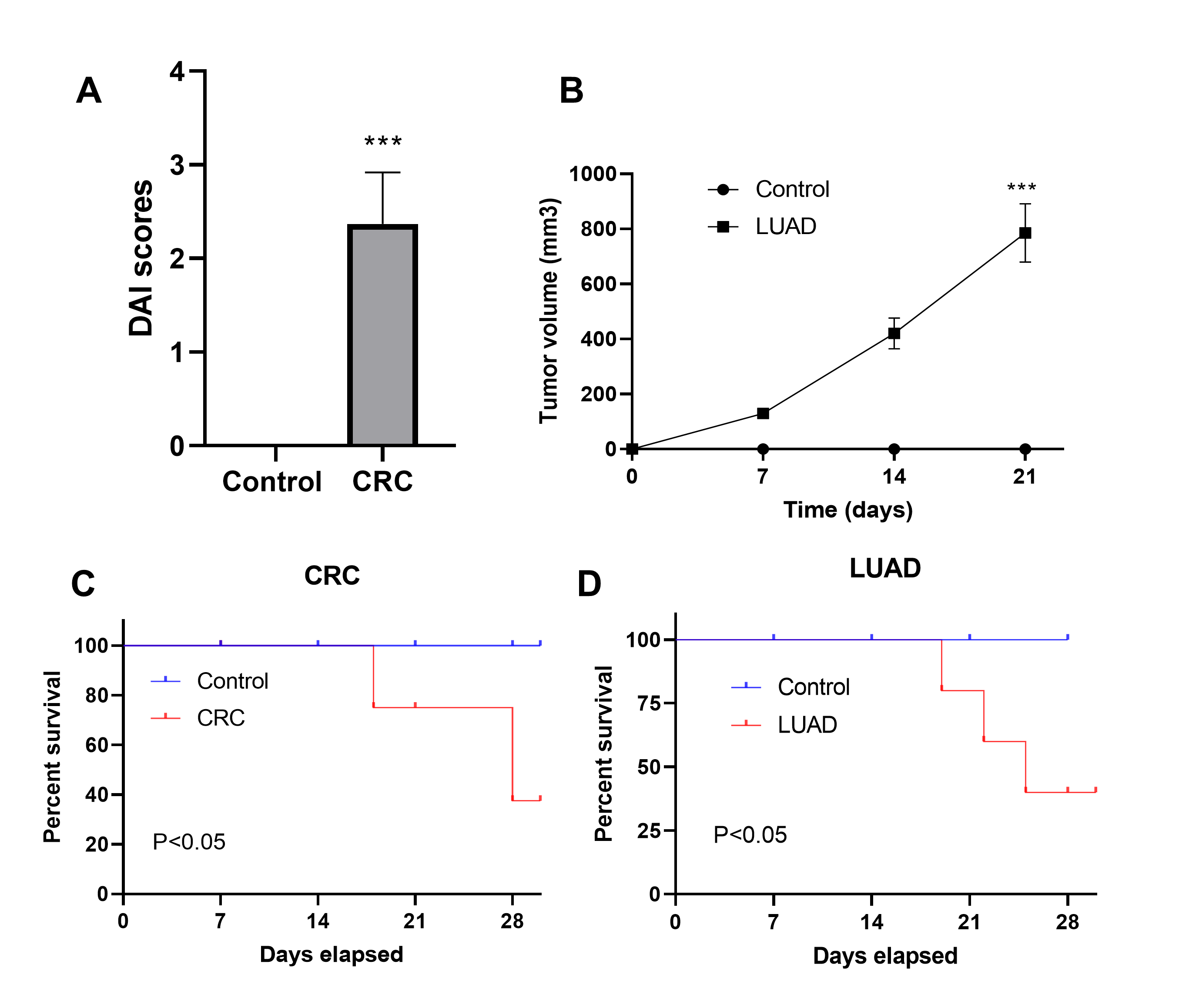


**Supplemental Fig. 2 CRC and LUAD mice model construction**

A, disease activity index of CRC mice model after AOM/DSS induction for 3 weeks. B, the change of tumor volume of LUAD in mice. C, the survival rate of CRC mice after 30 days. D, the survival rate of LUAD mice. ***P<0.001, compared with control group.
