# Supplementary figures and images for: Investigation of biomarkers and associated molecular mechanism shared between colorectal cancer and lung adenocarcinoma
Source: Discov Oncol. 2025 Aug 12;16:1540. doi: 10.1007/s12672-025-03240-5 (PMC12343392; doi:10.1007/s12672-025-03240-5)

GAPDH-1


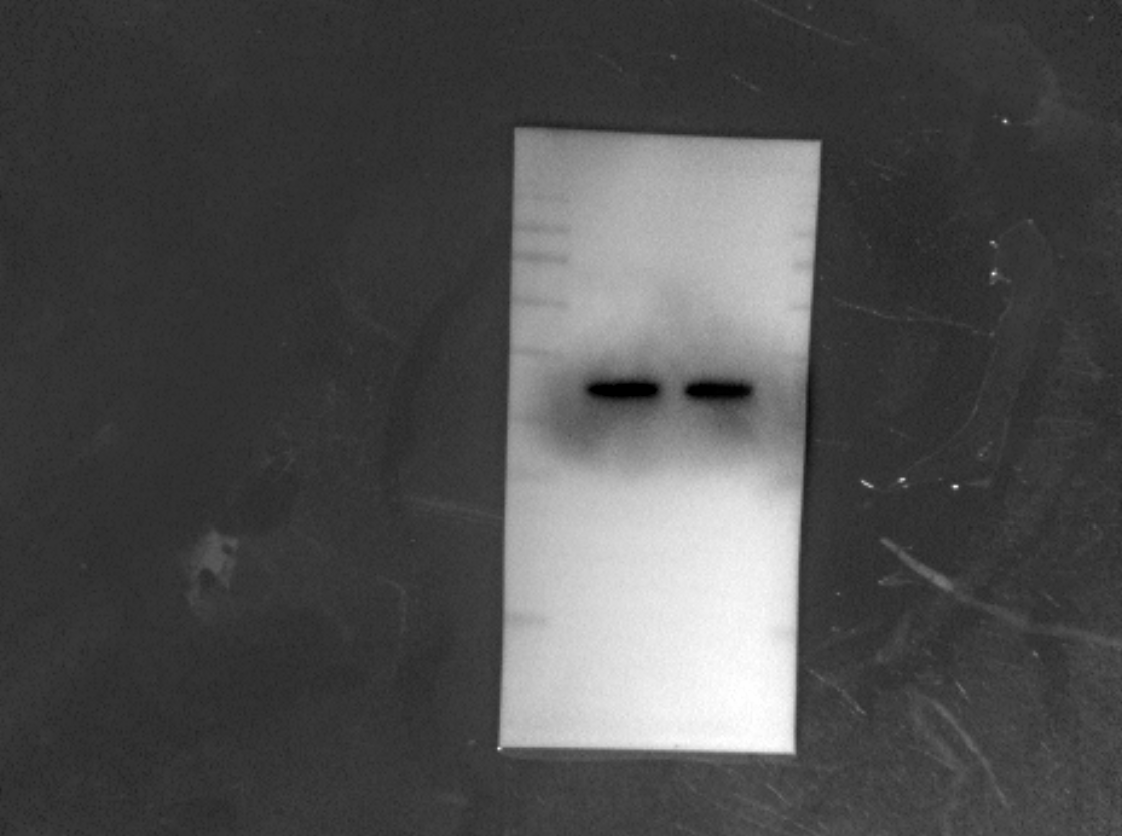


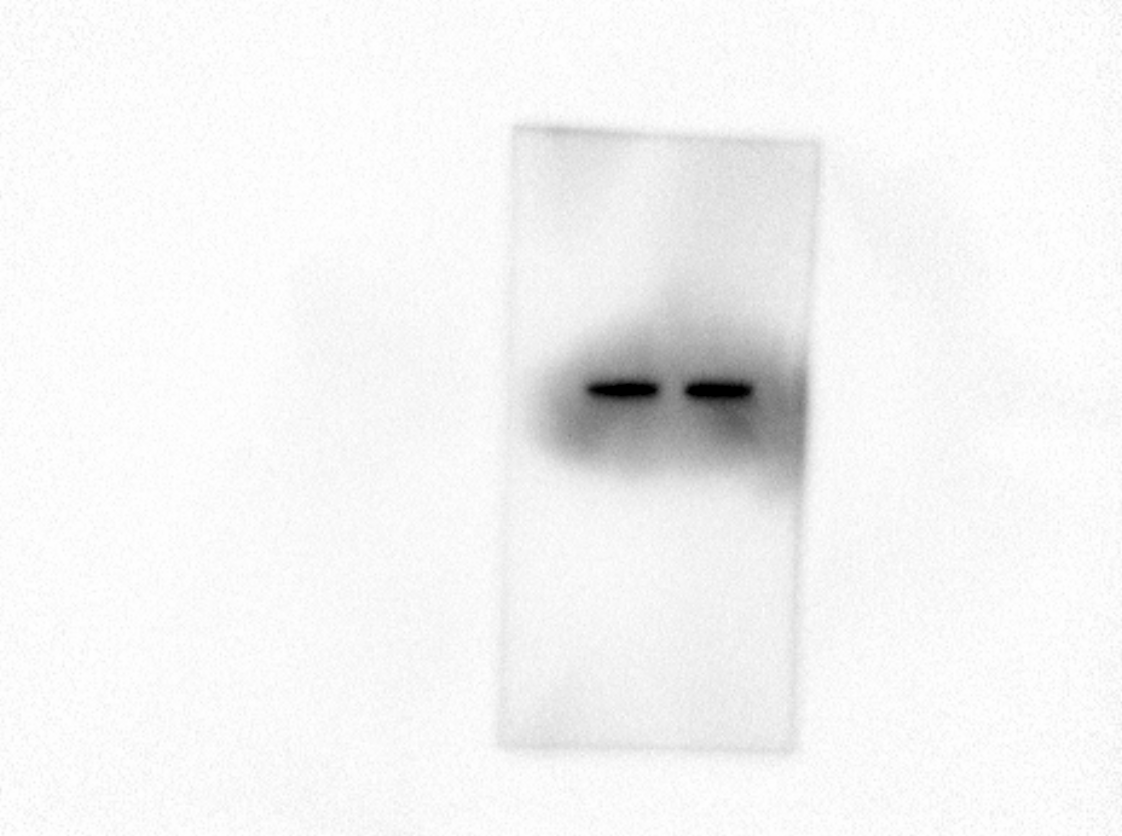


GAPDH-2


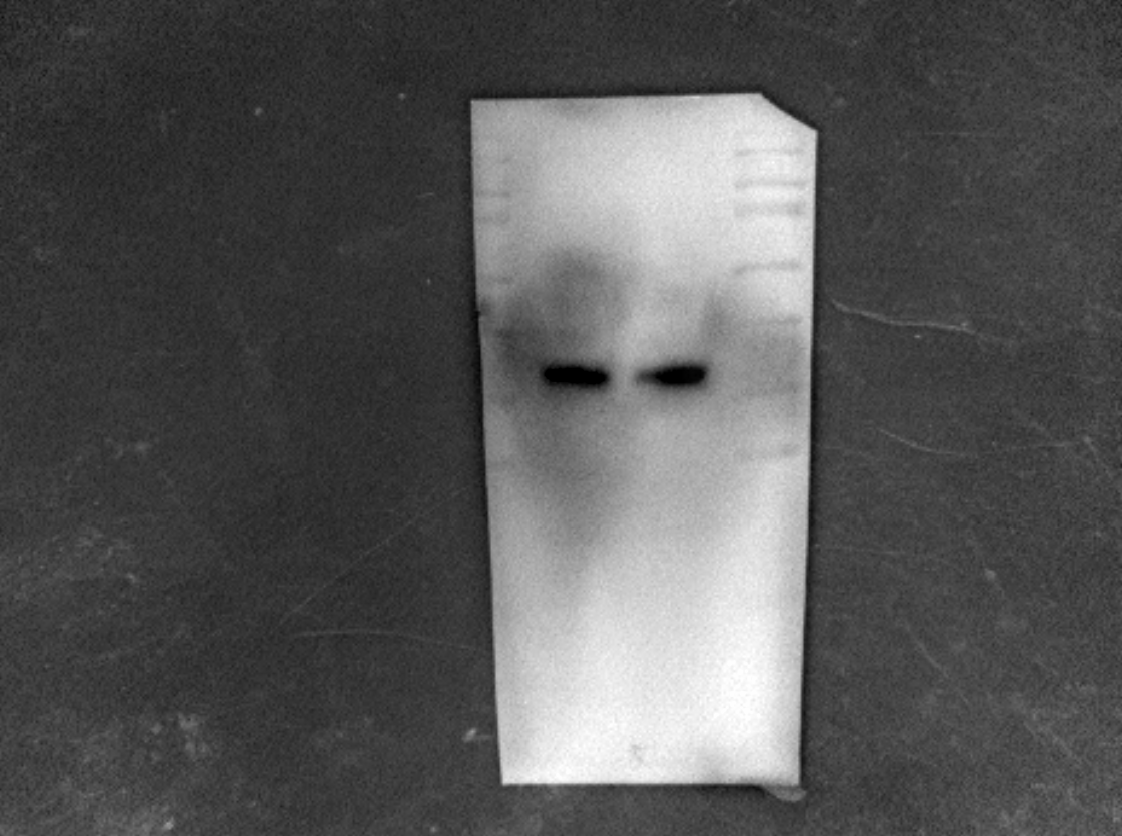

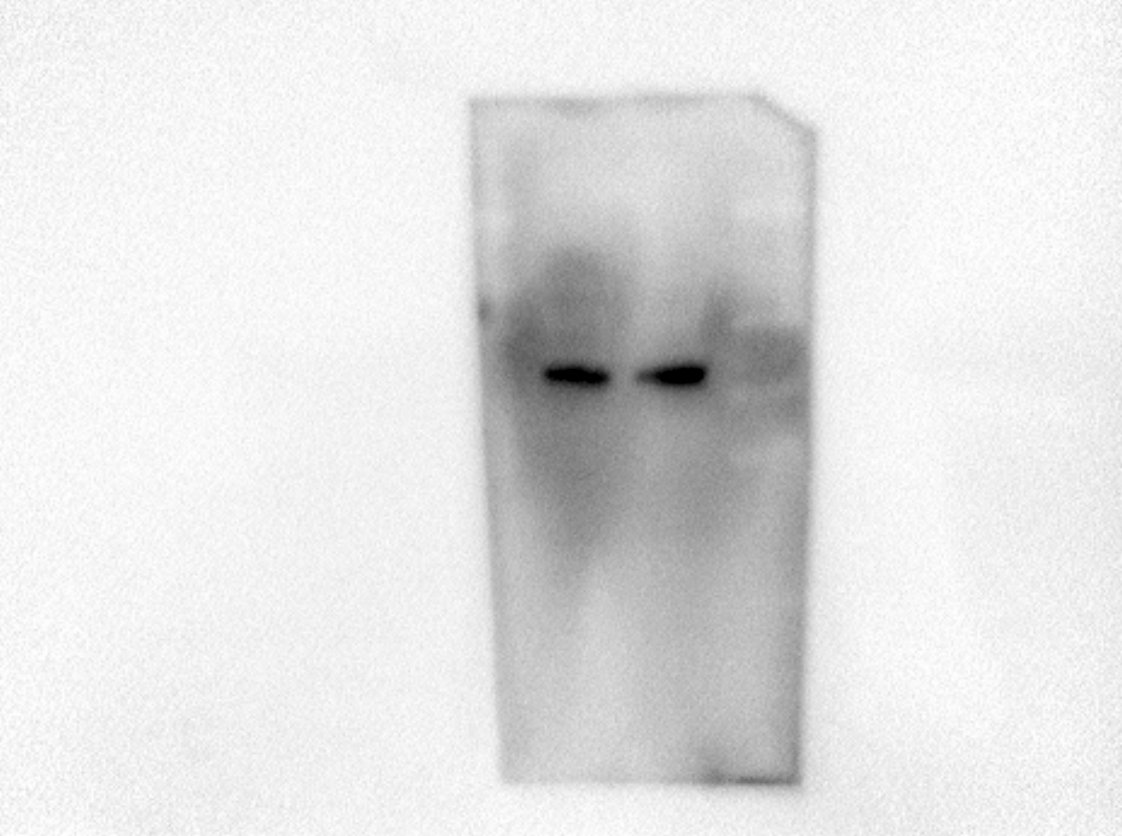


GAPDH-3


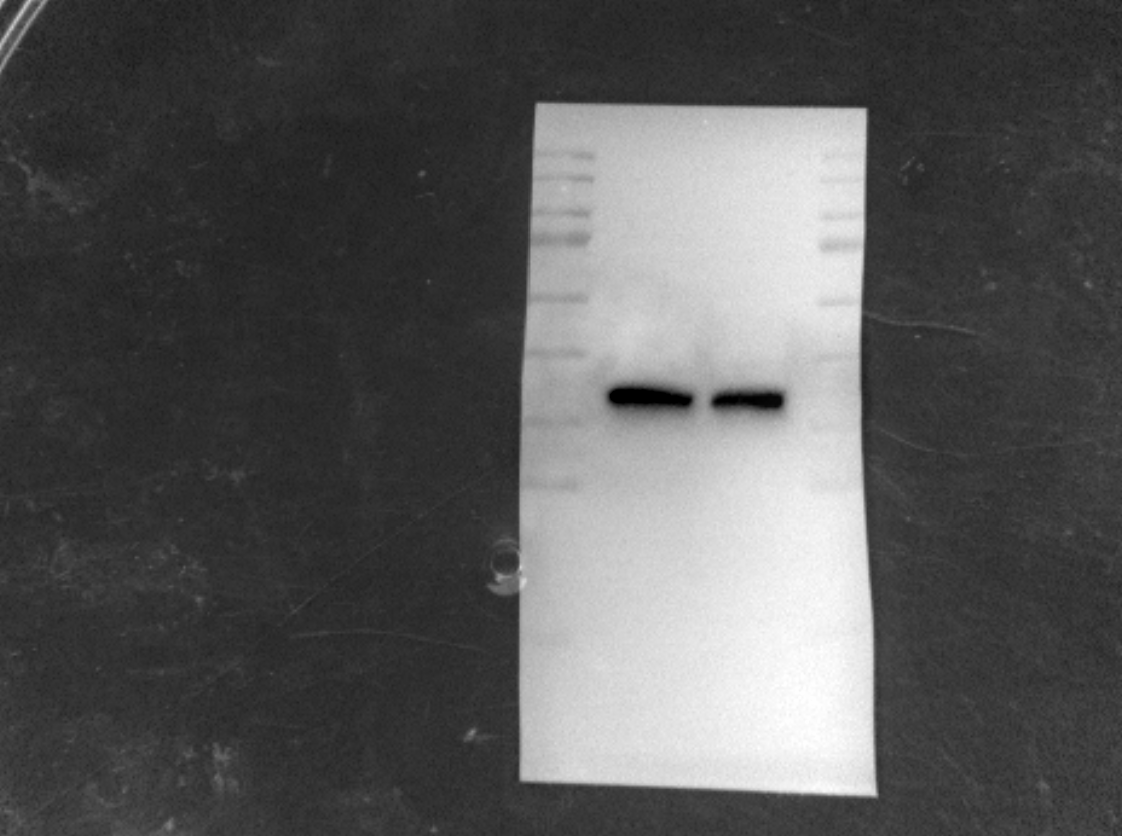


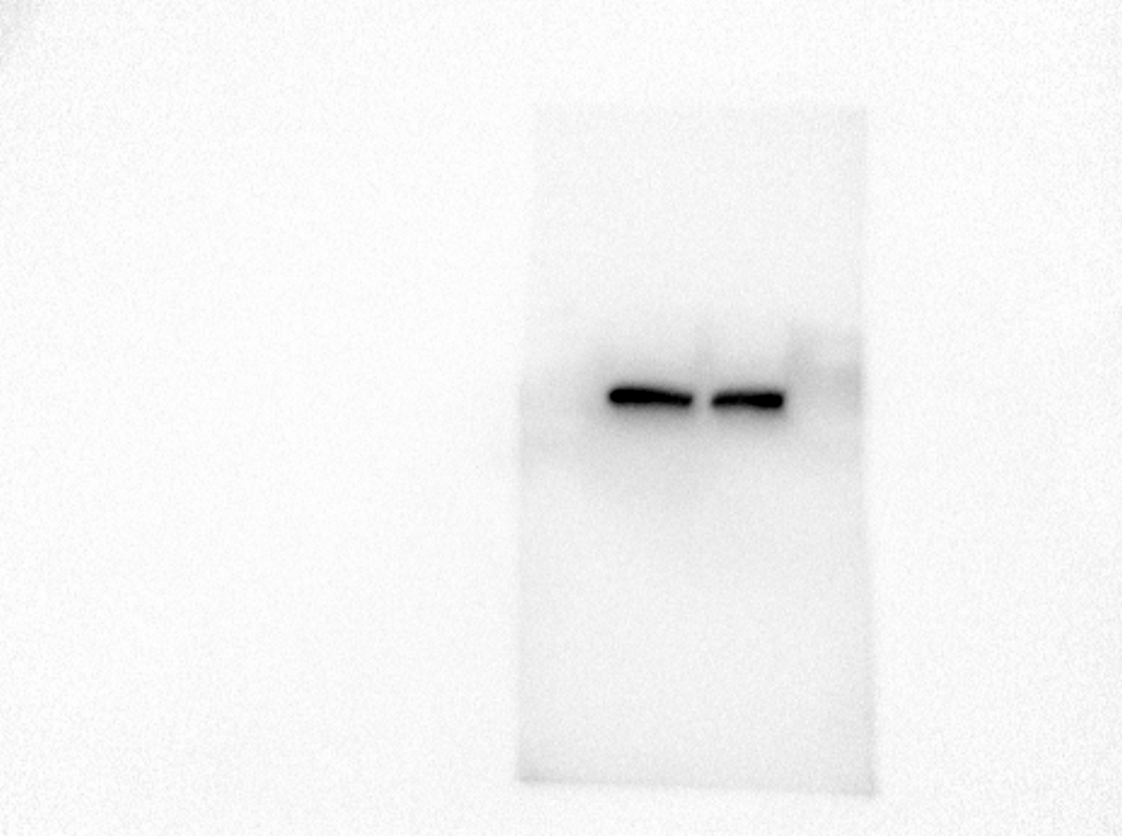


GPD1L-1


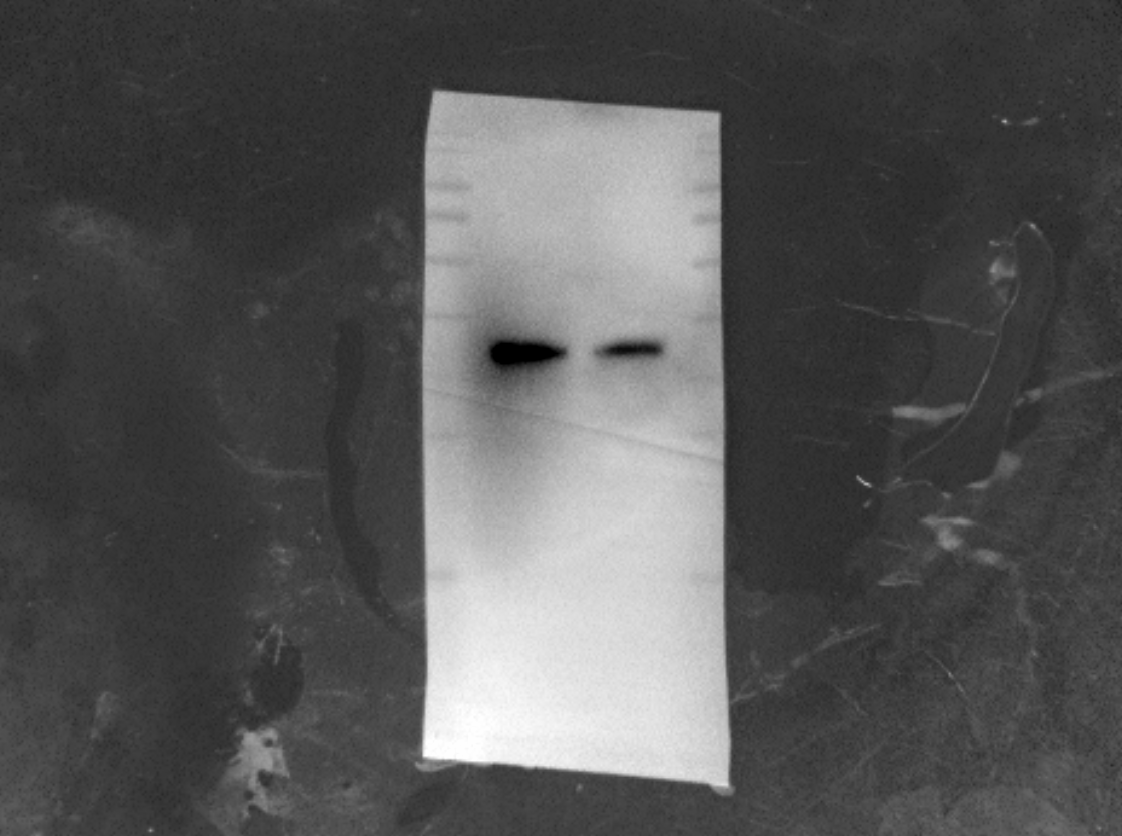

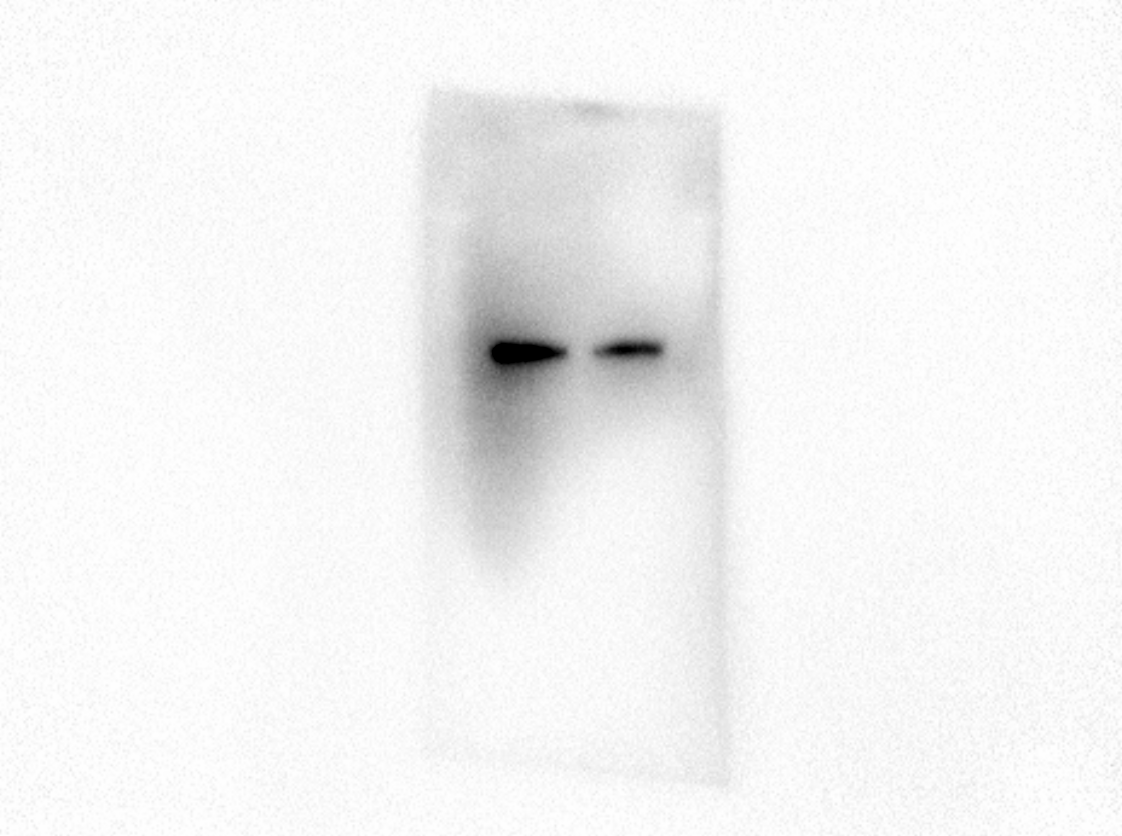


GPD1L-2


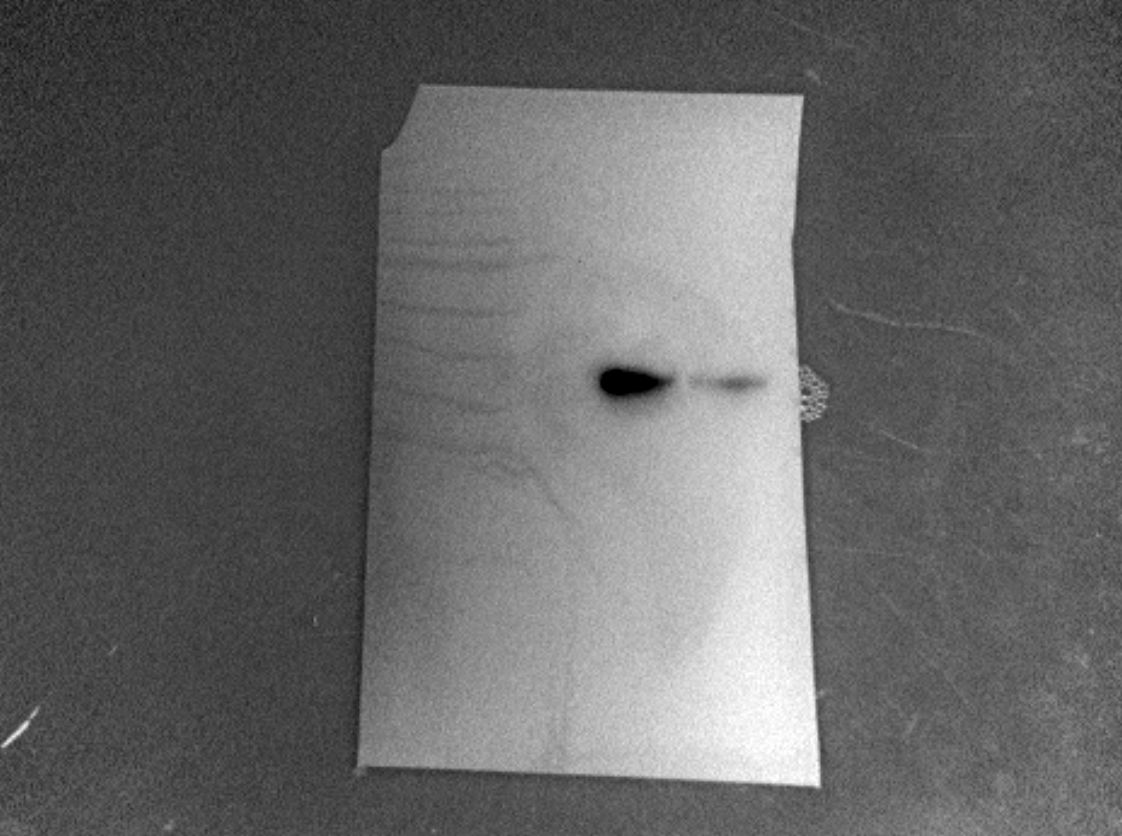

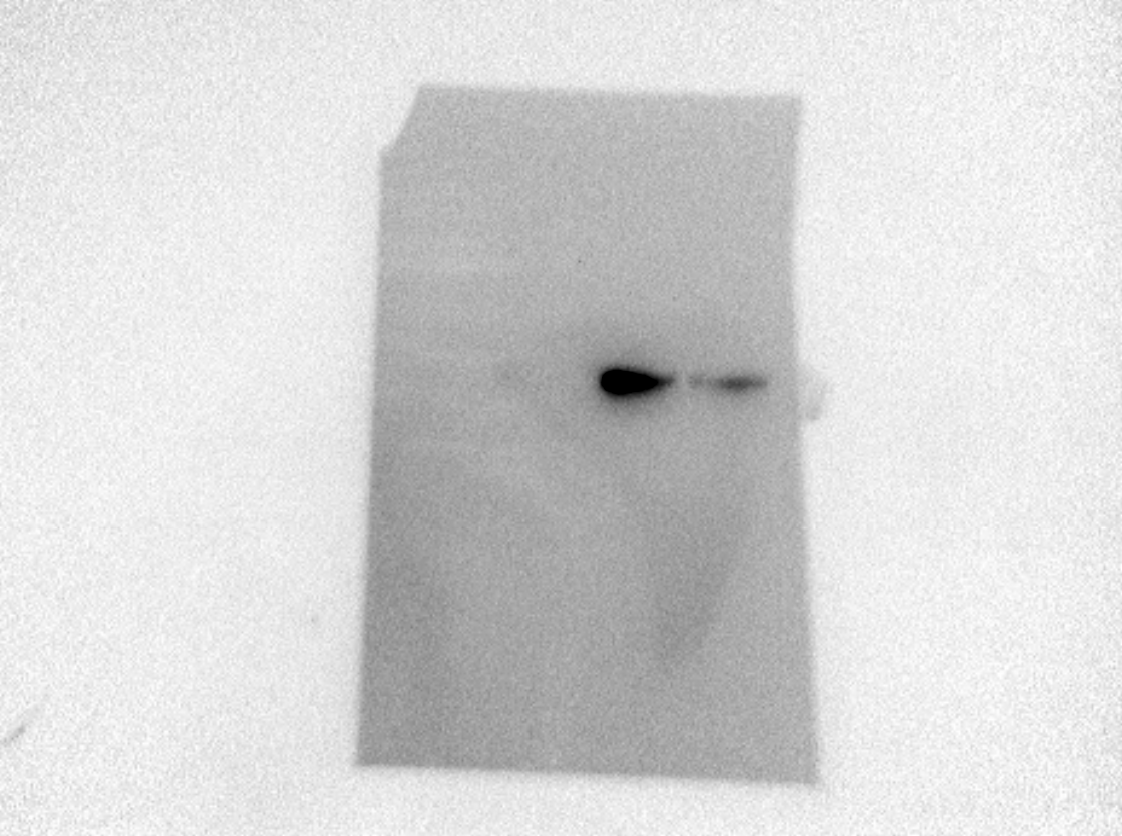


GPD1L-3


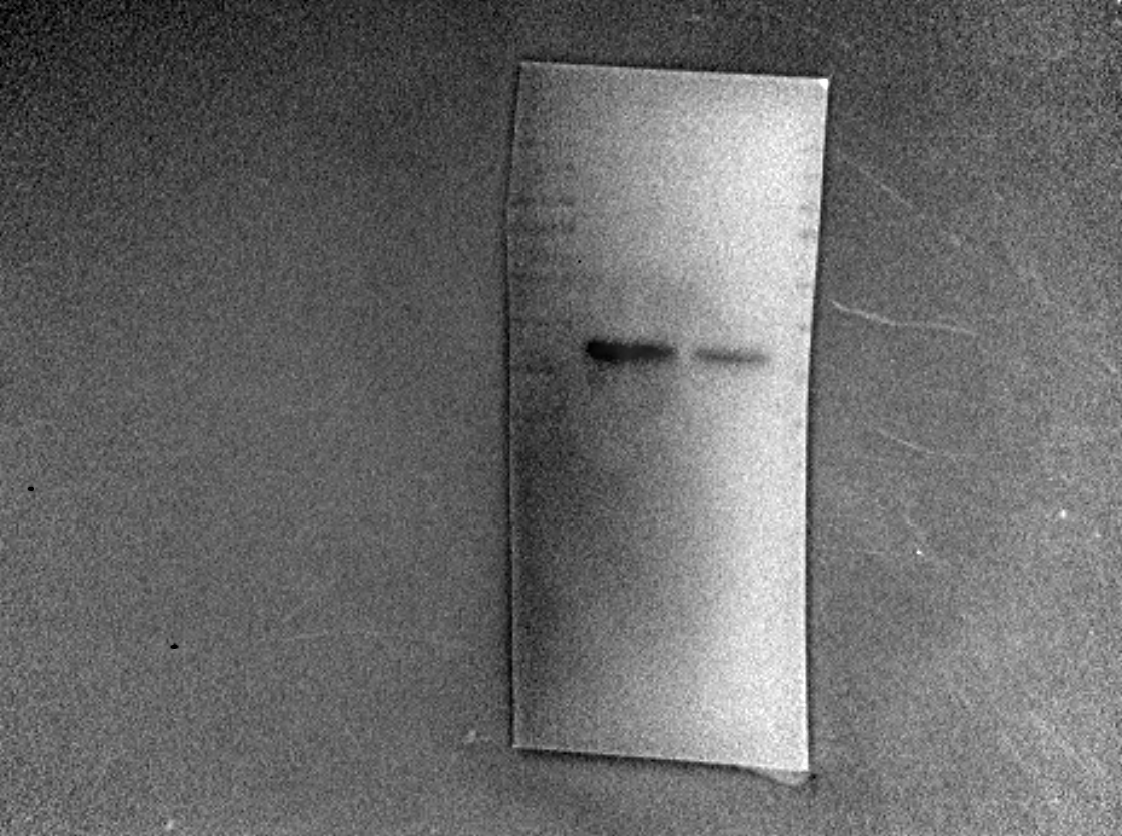

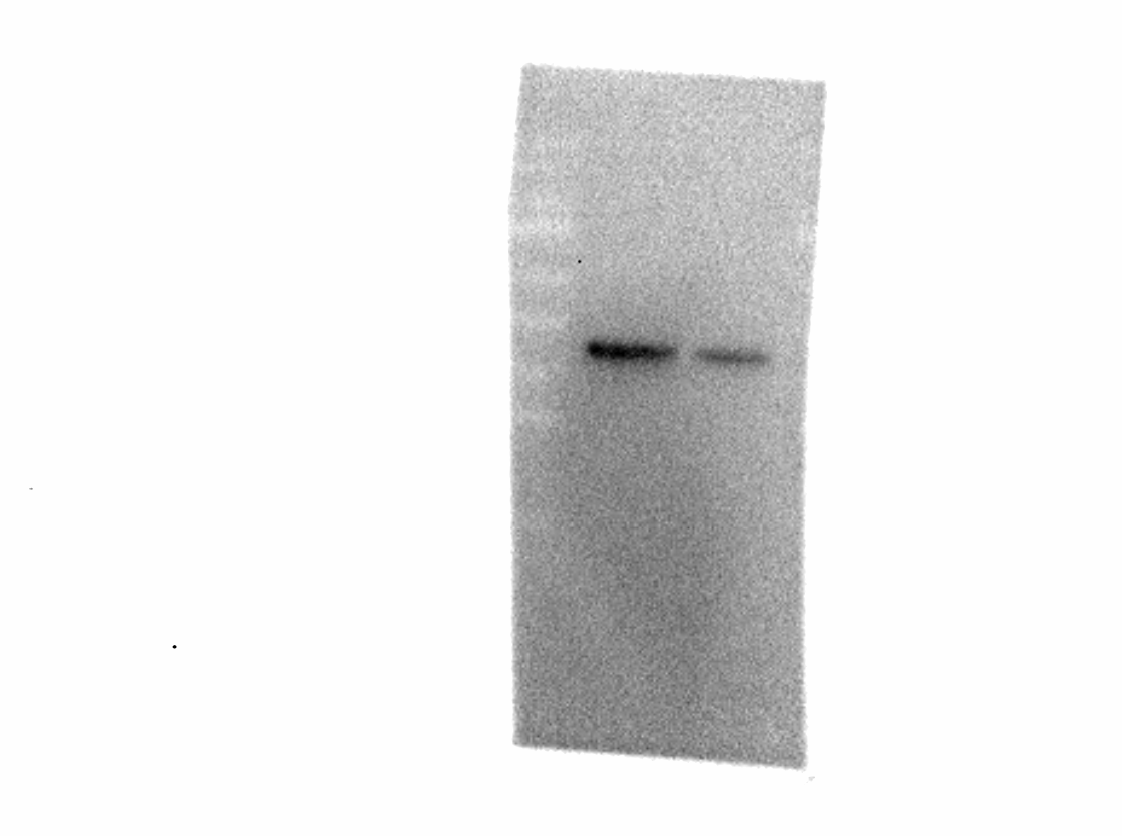


HSPA6-1


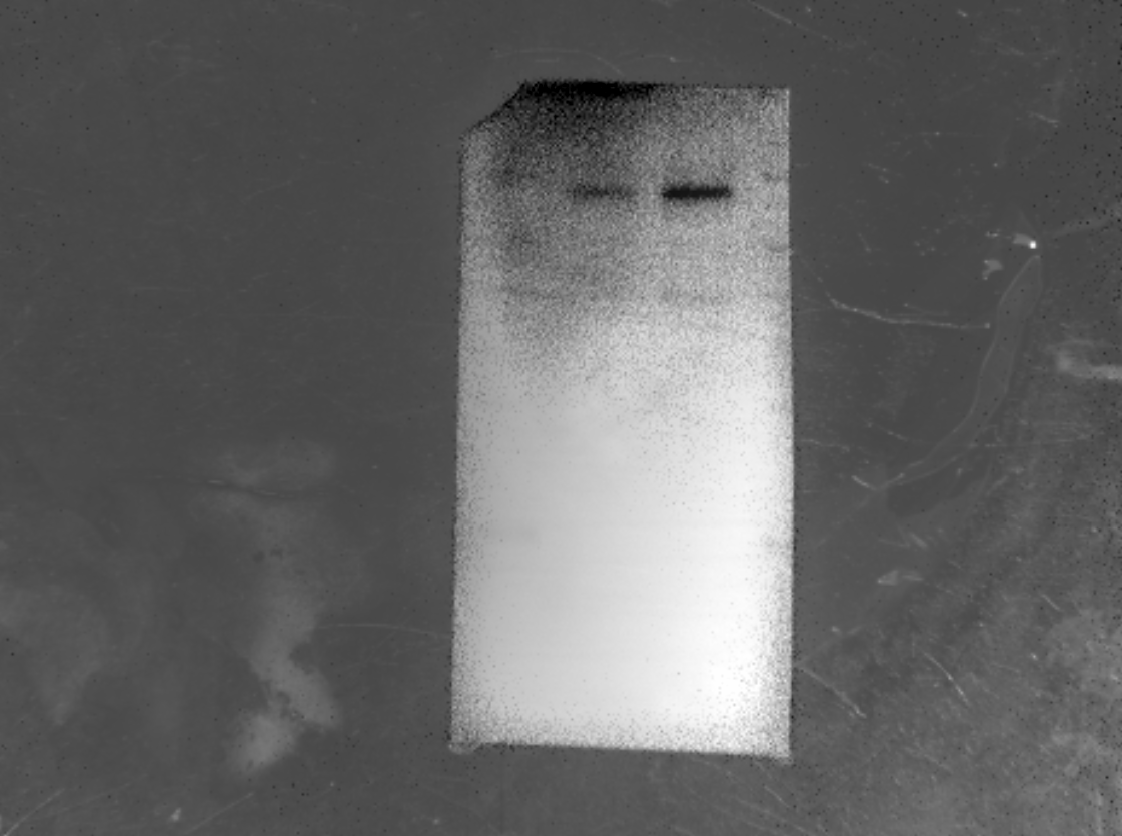

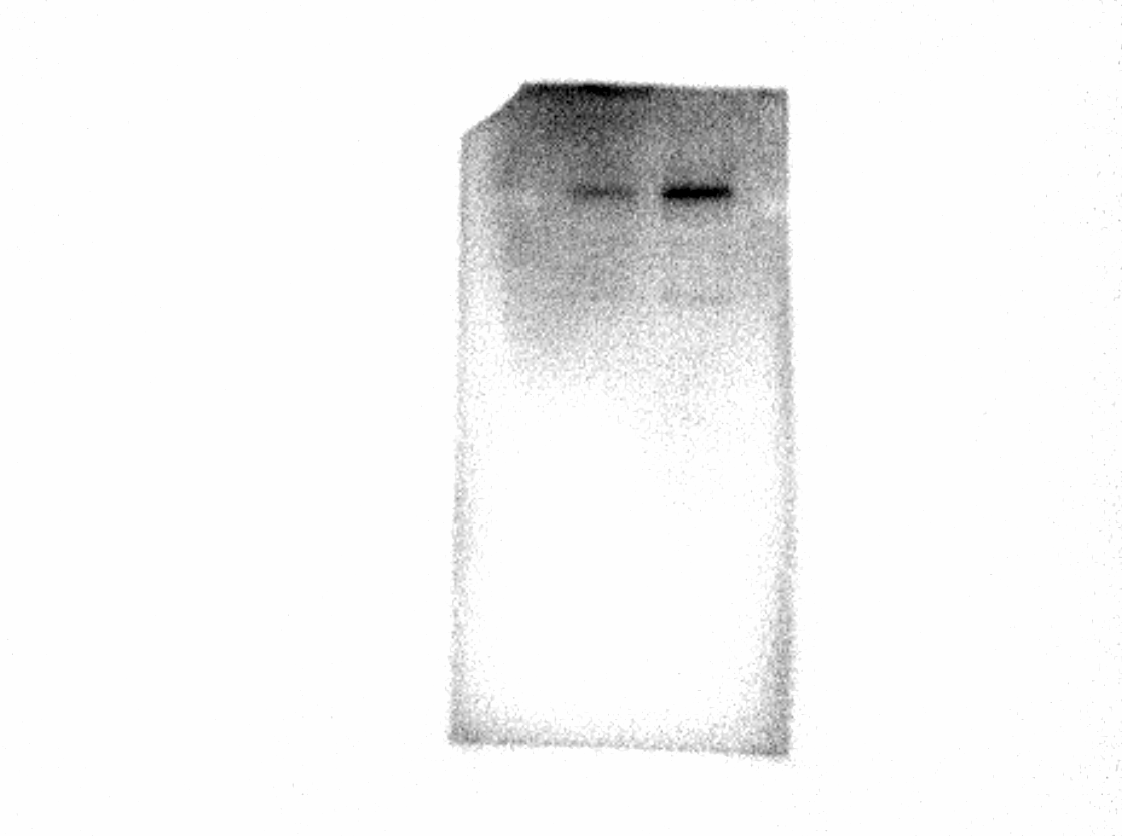


HSPA6-2


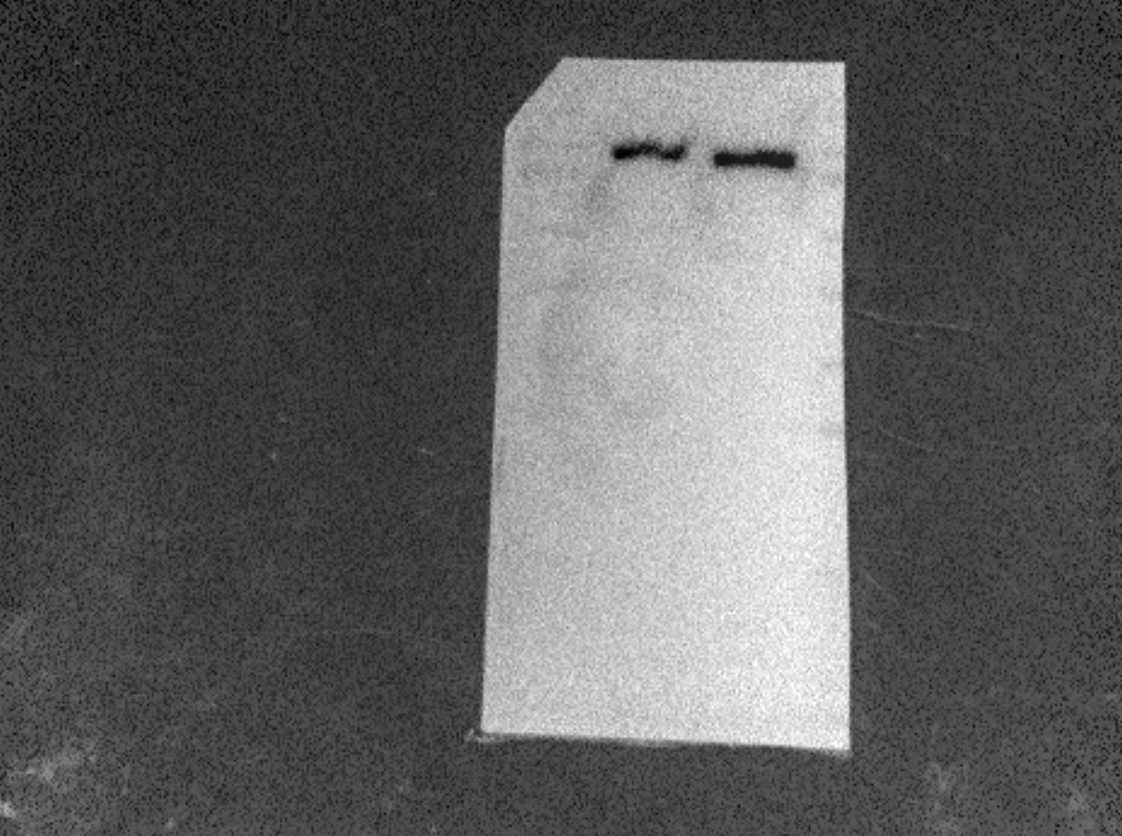


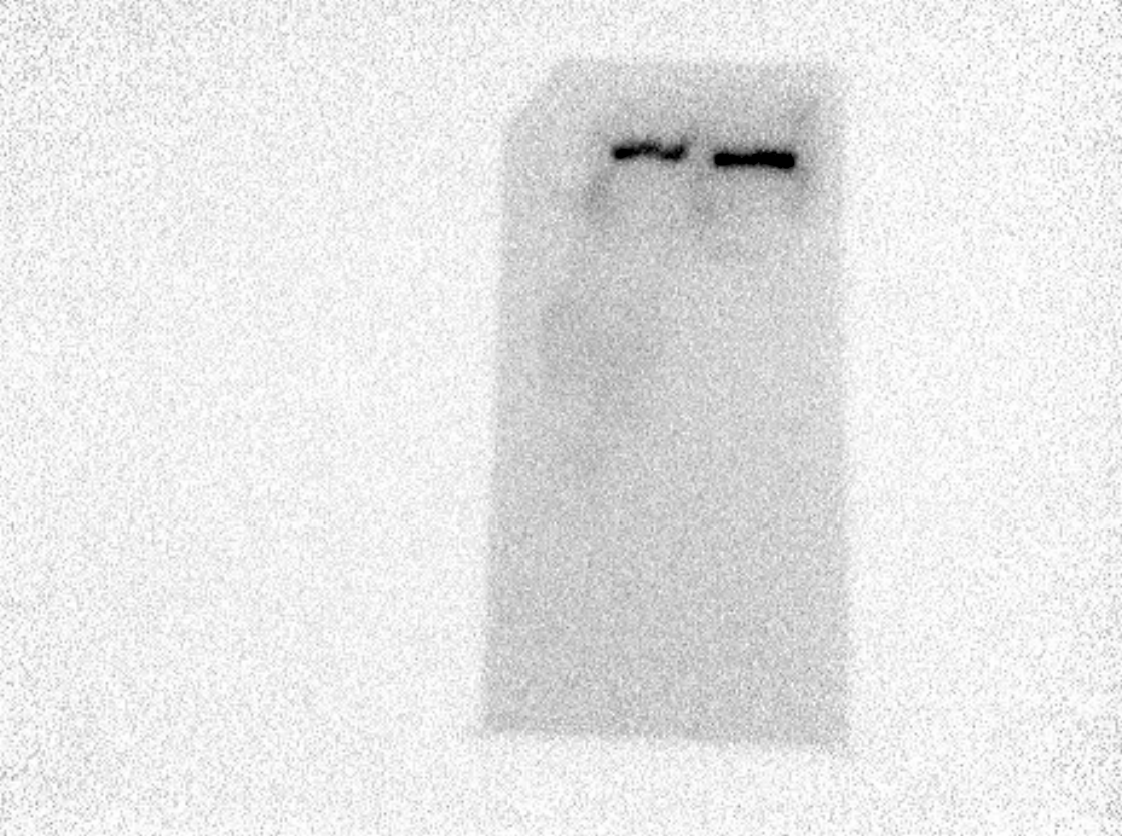


HSPA6-3


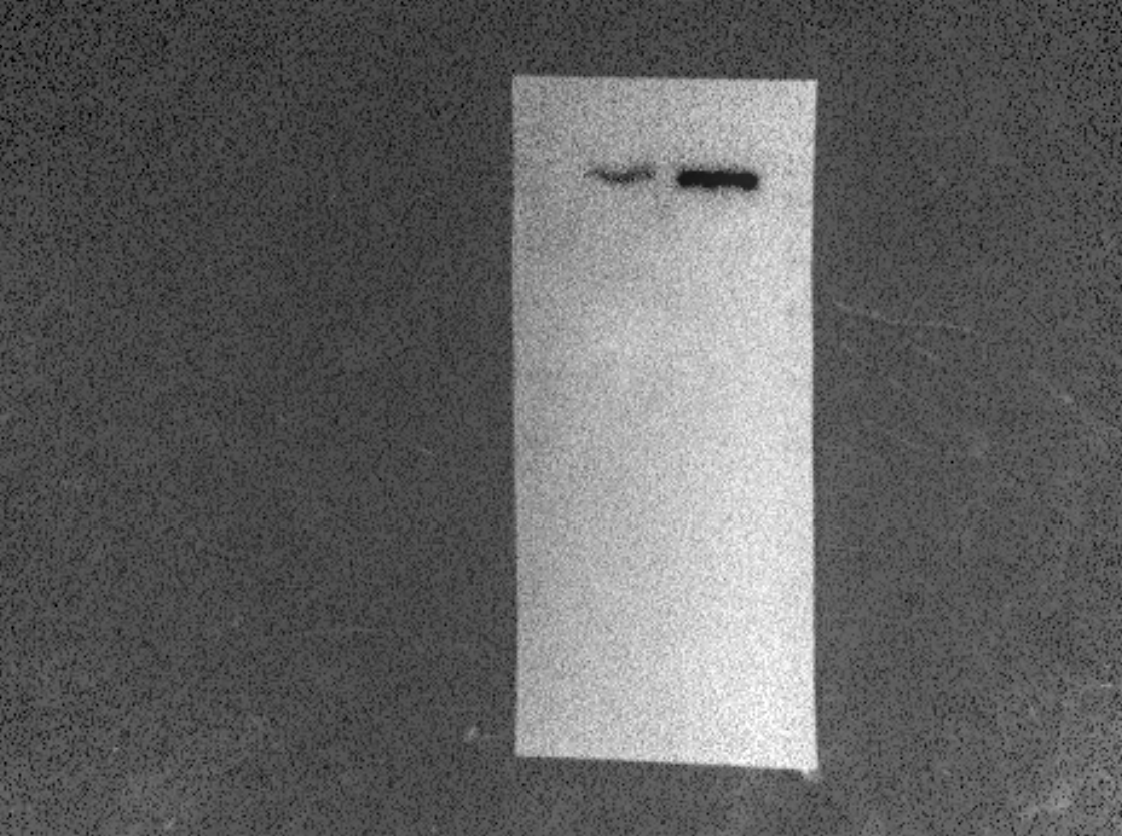

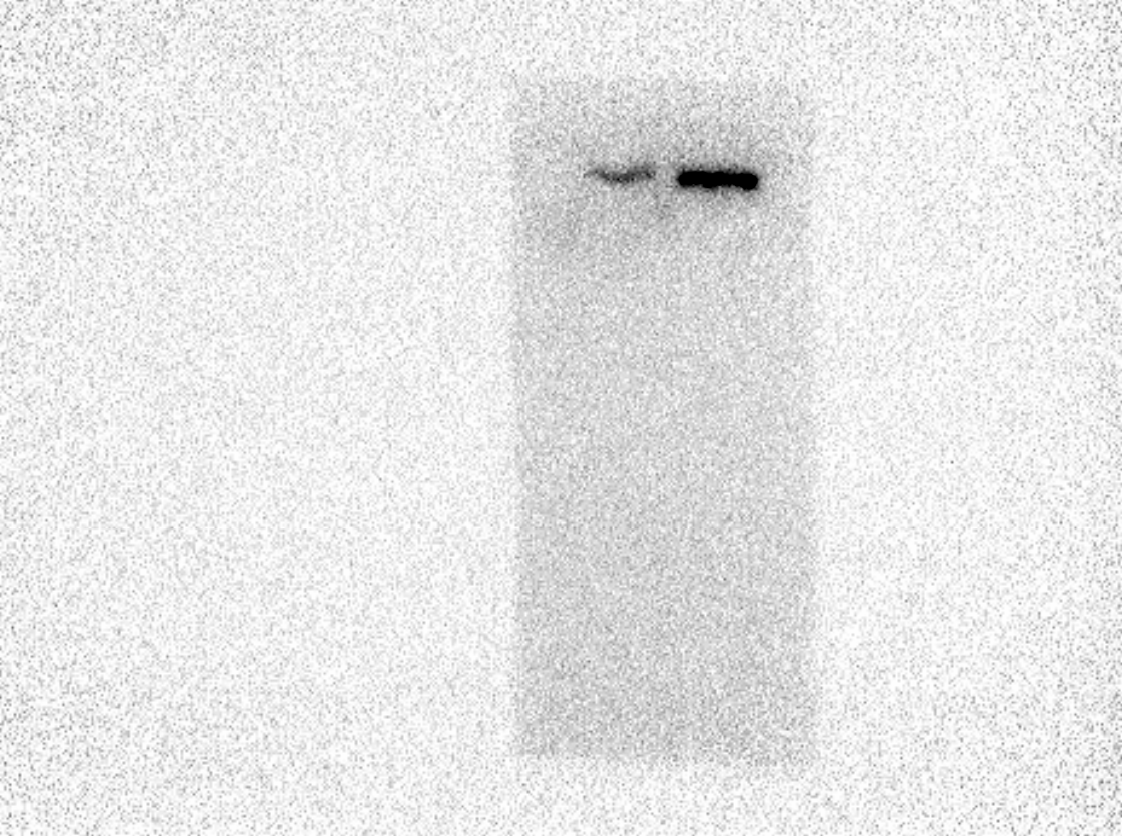


NOCTCH3-1


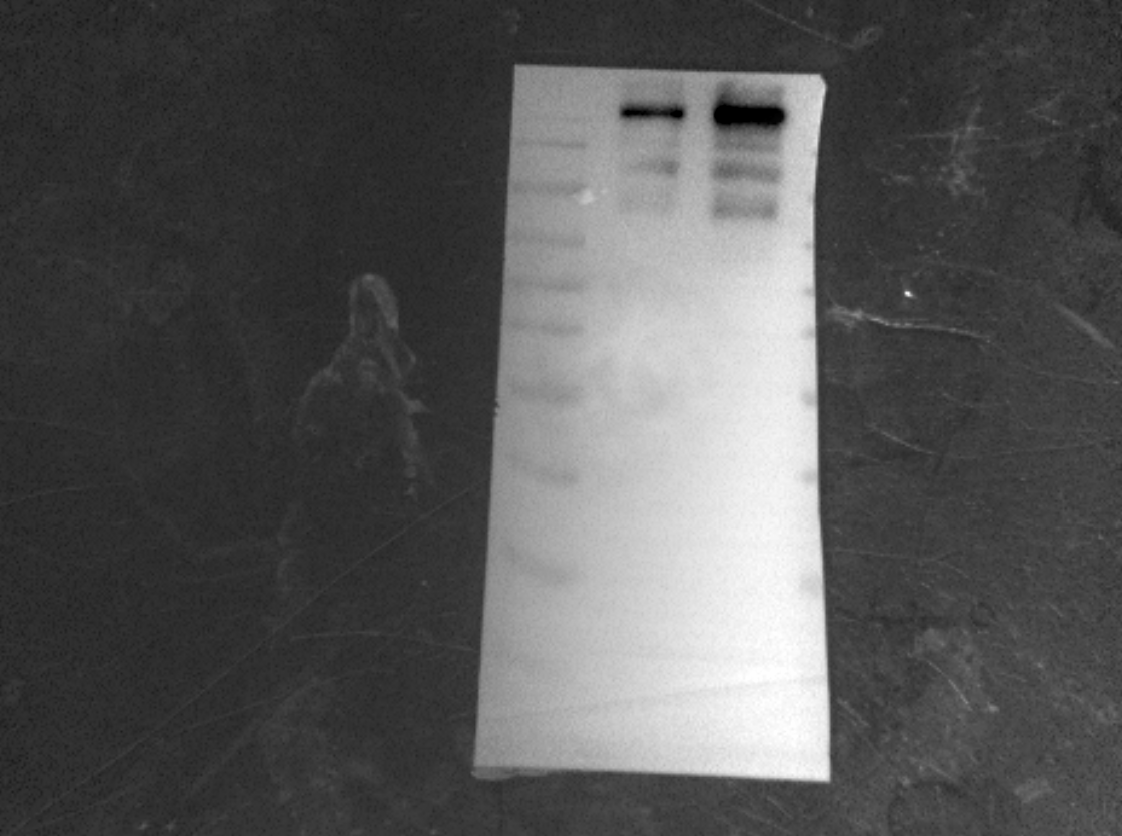


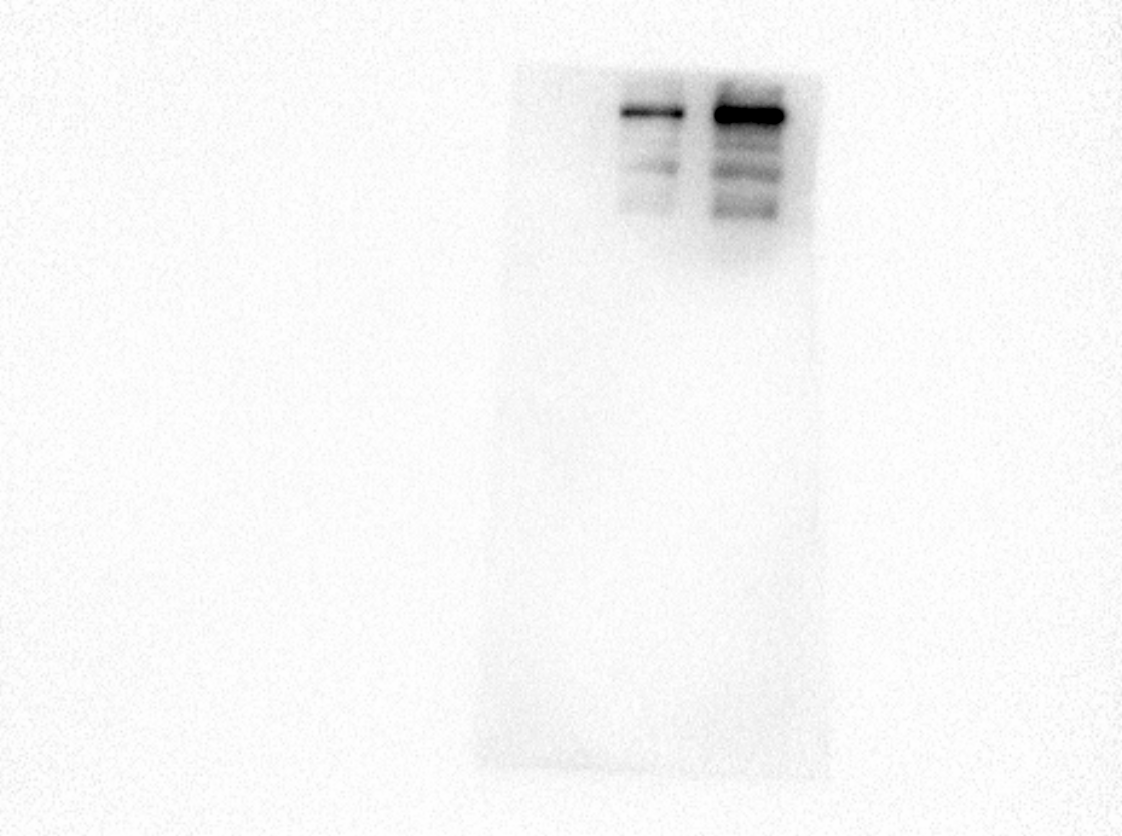


NOCTCH3-2


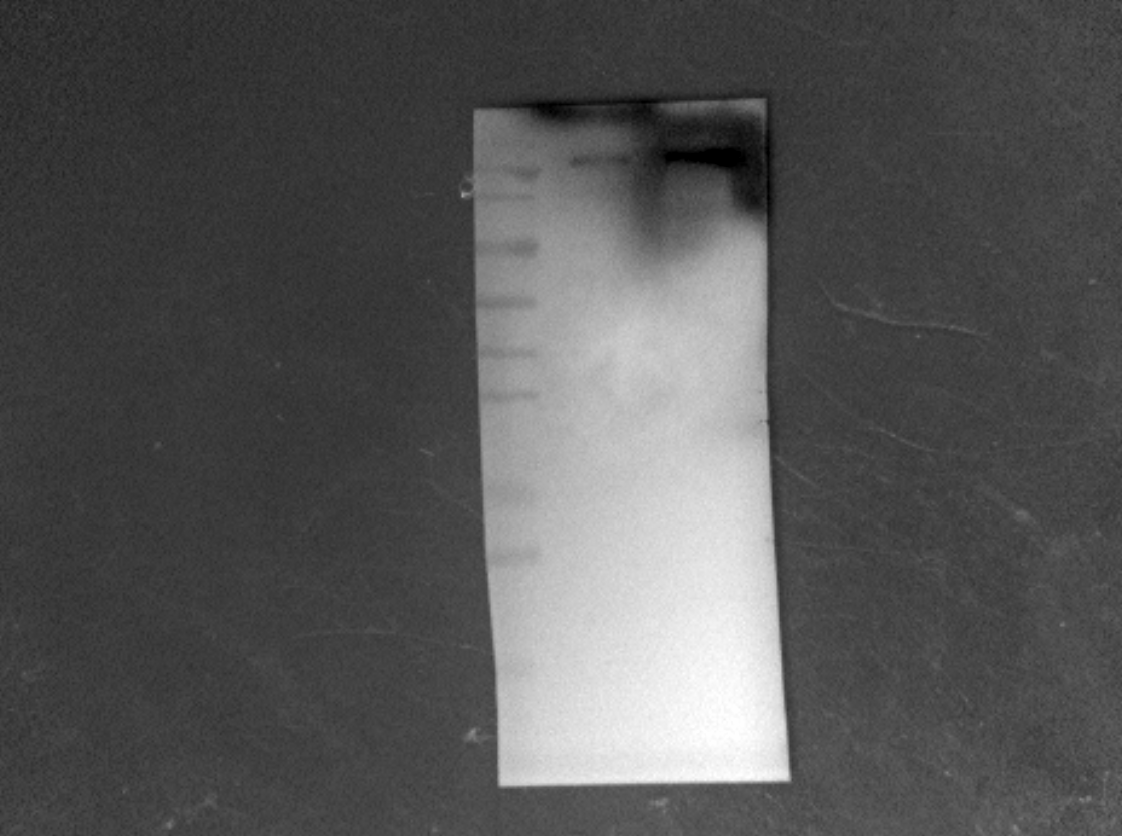


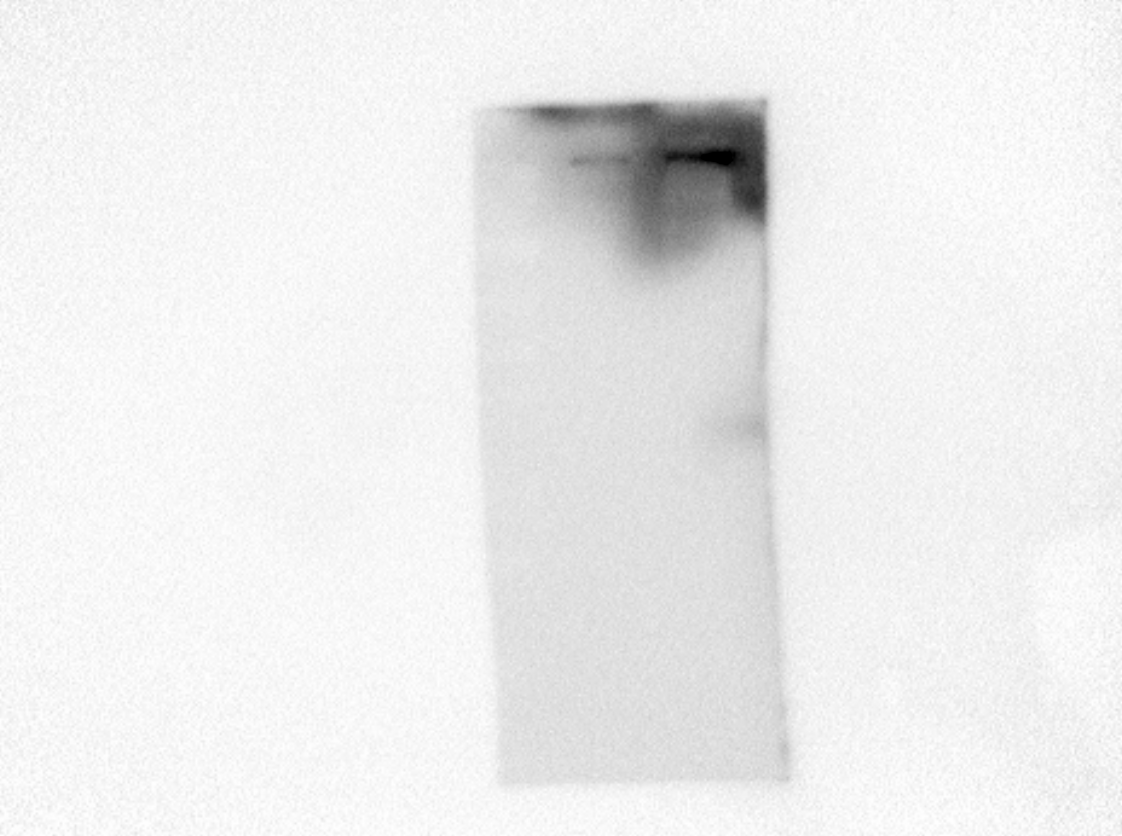


NOTCH3-3


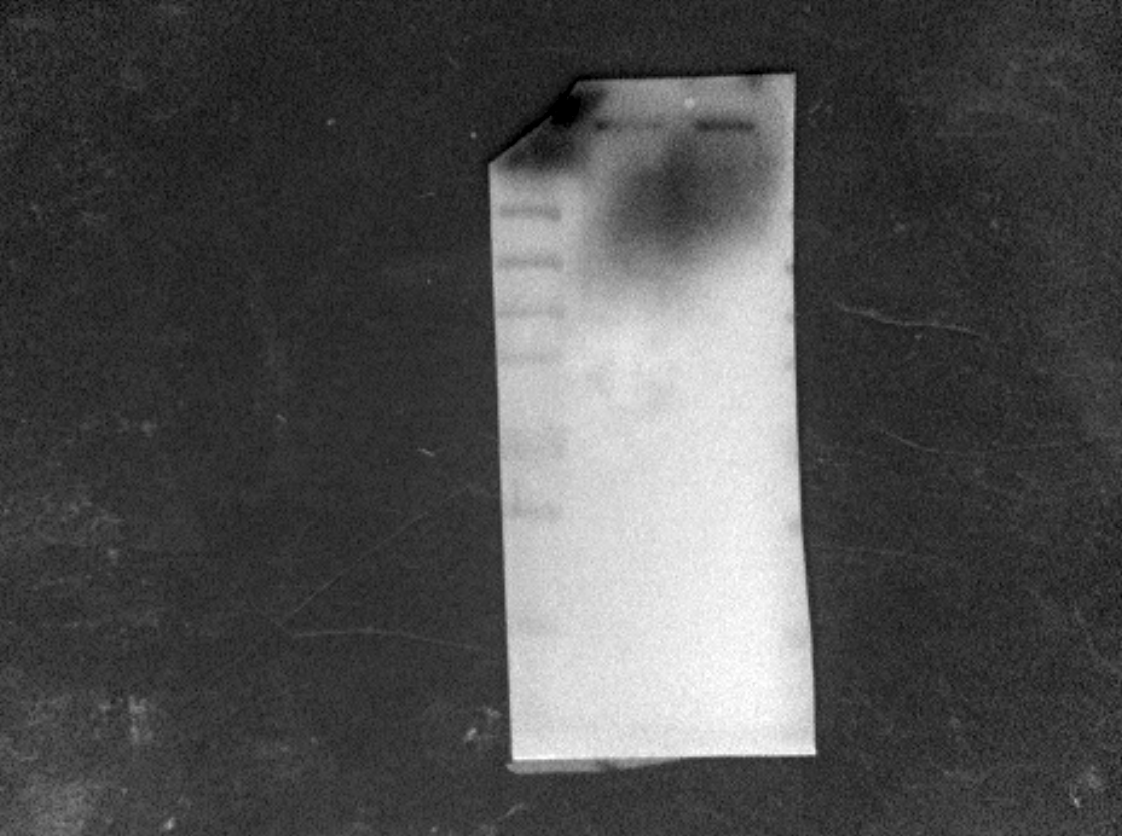


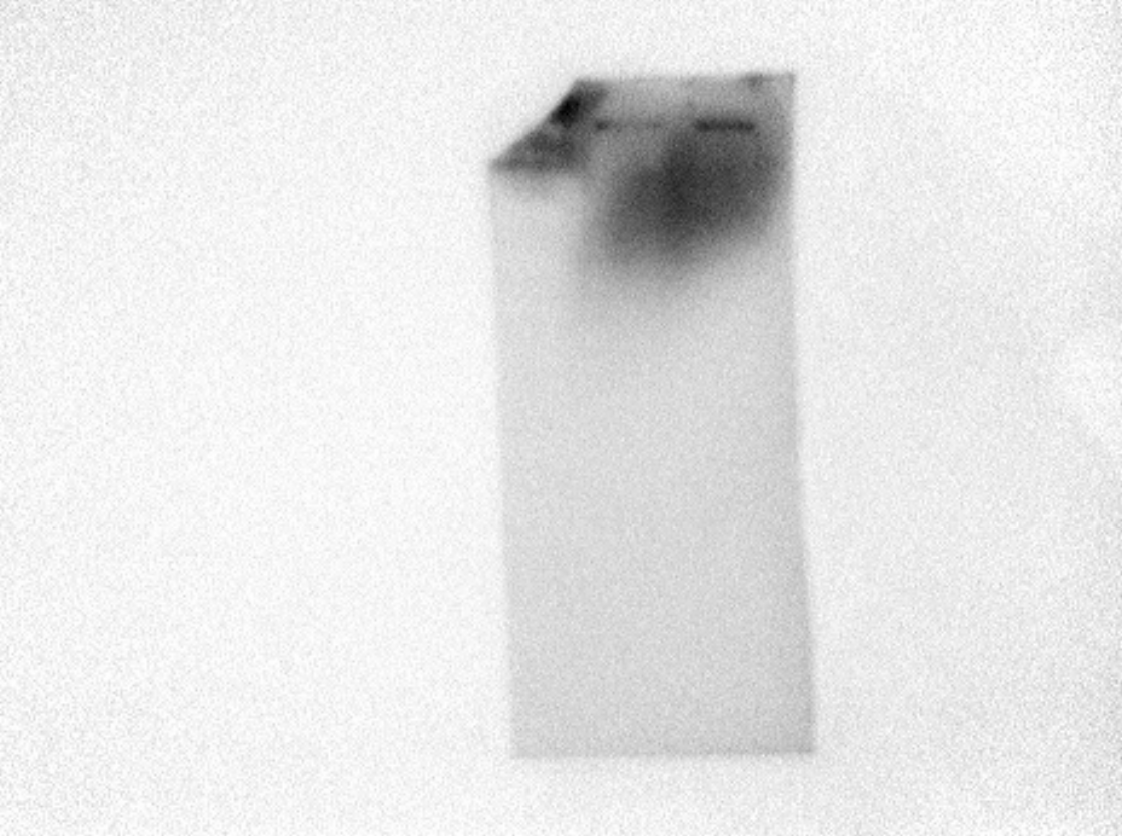


PKP2-1


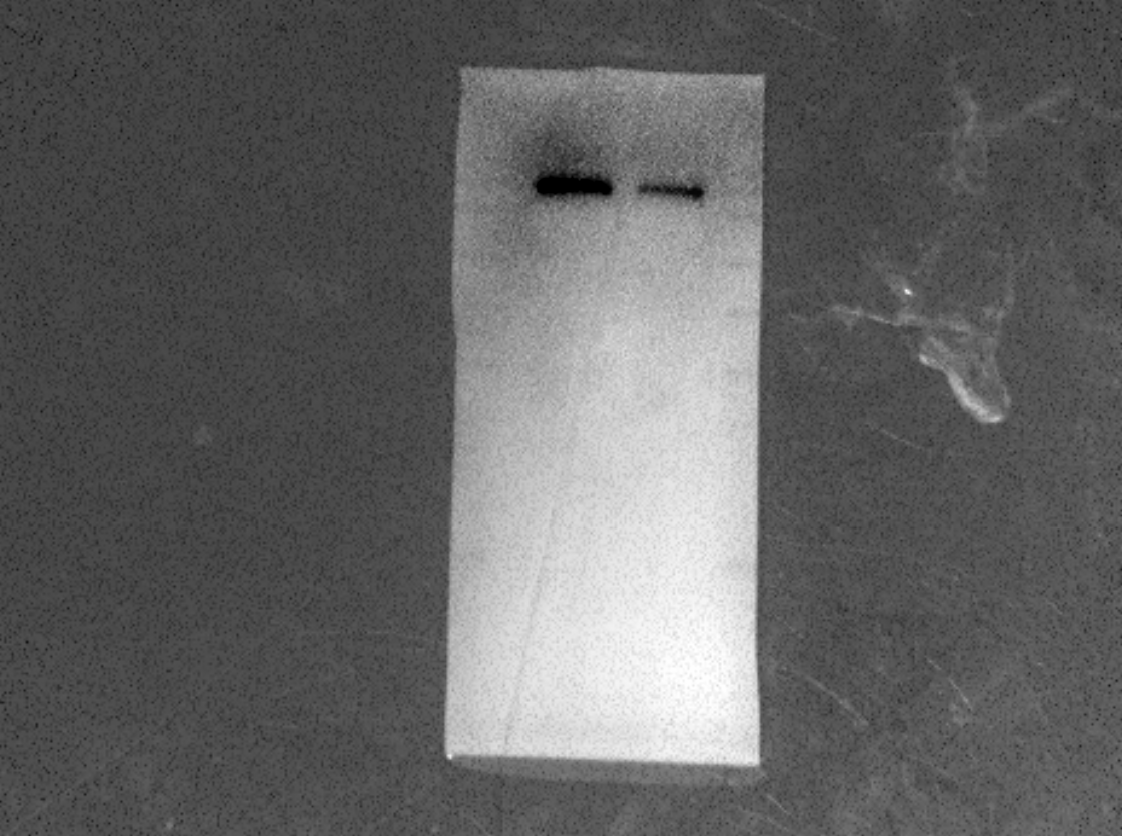


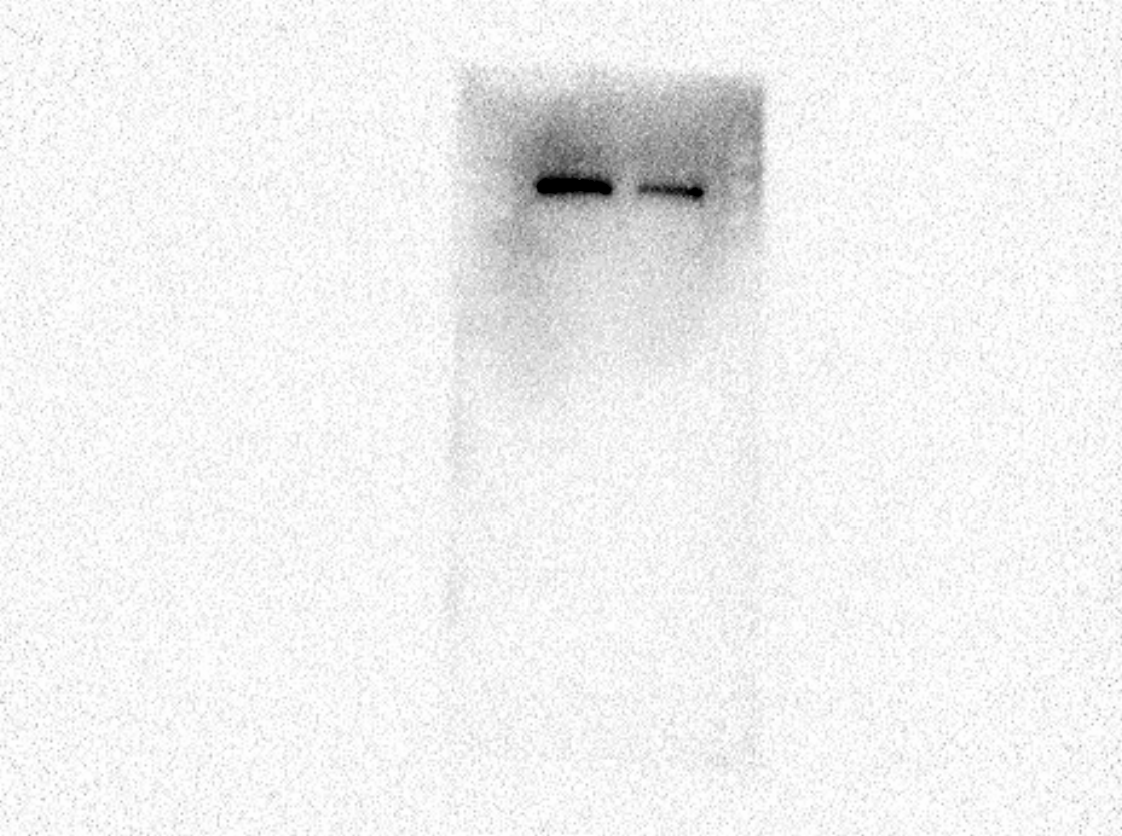


PKP2-2


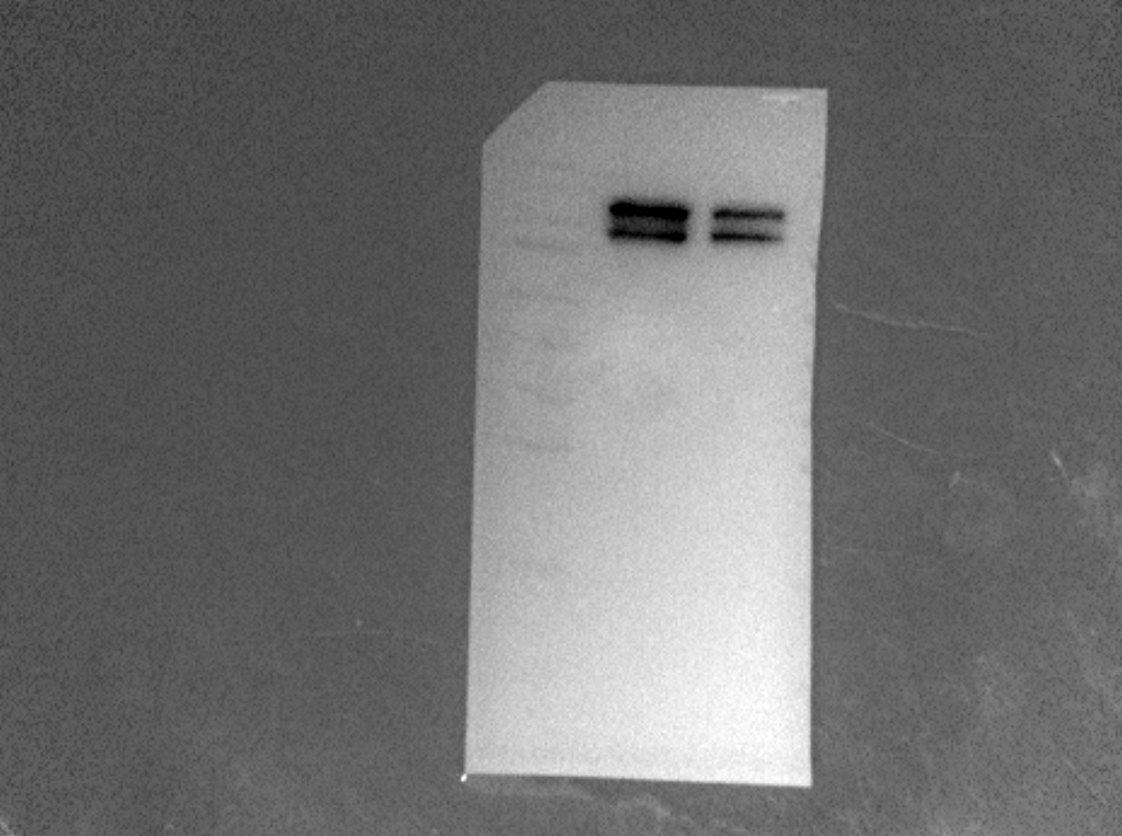


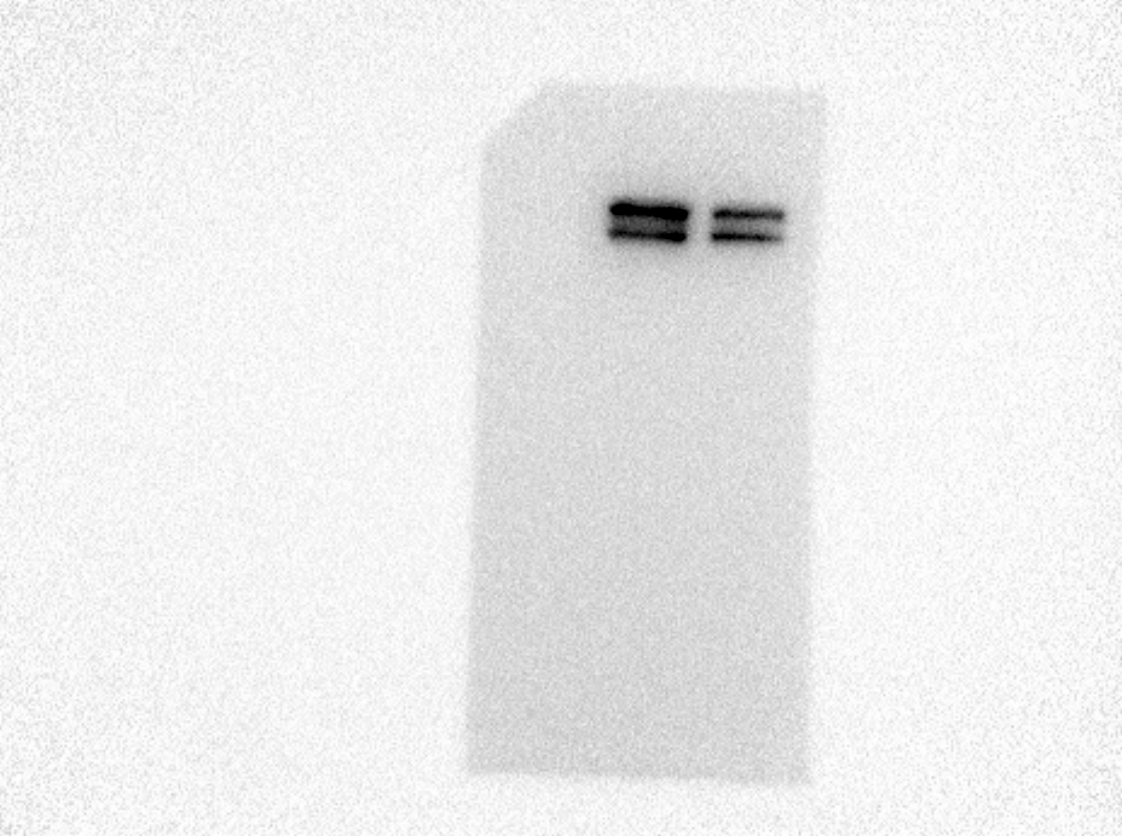


PKP2-3


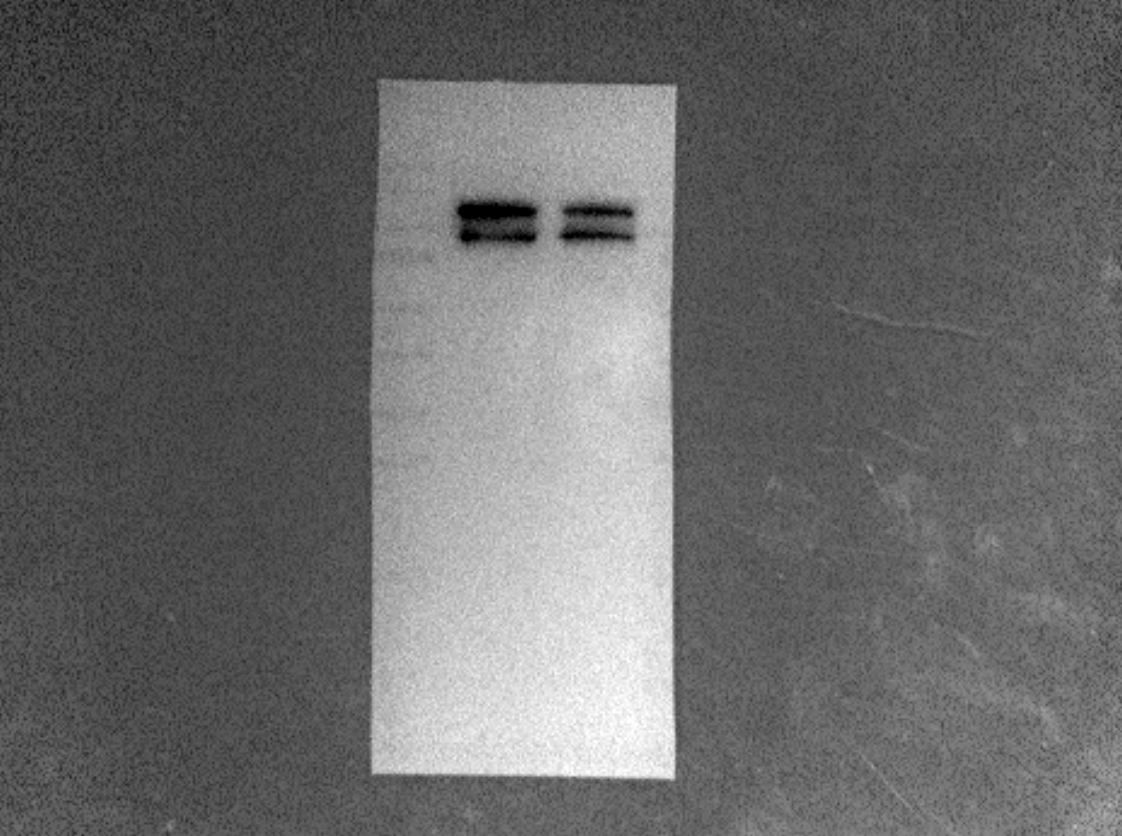


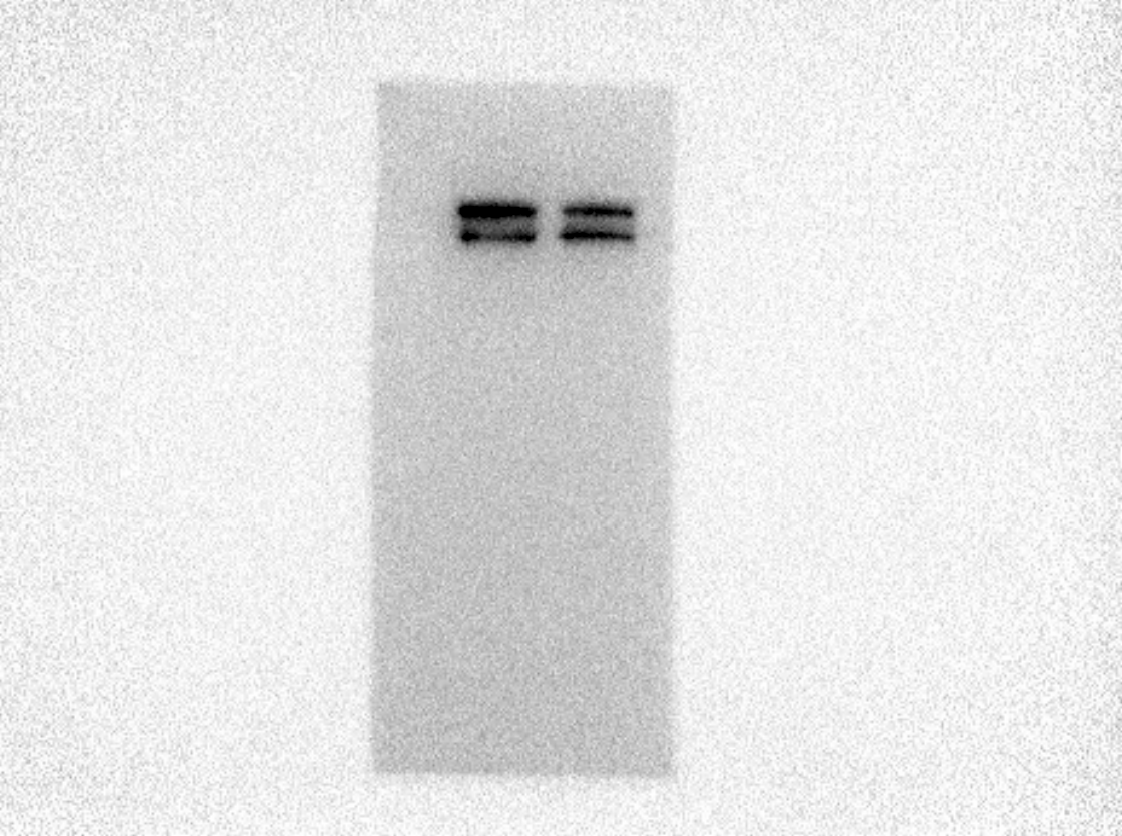


SMAD9-1


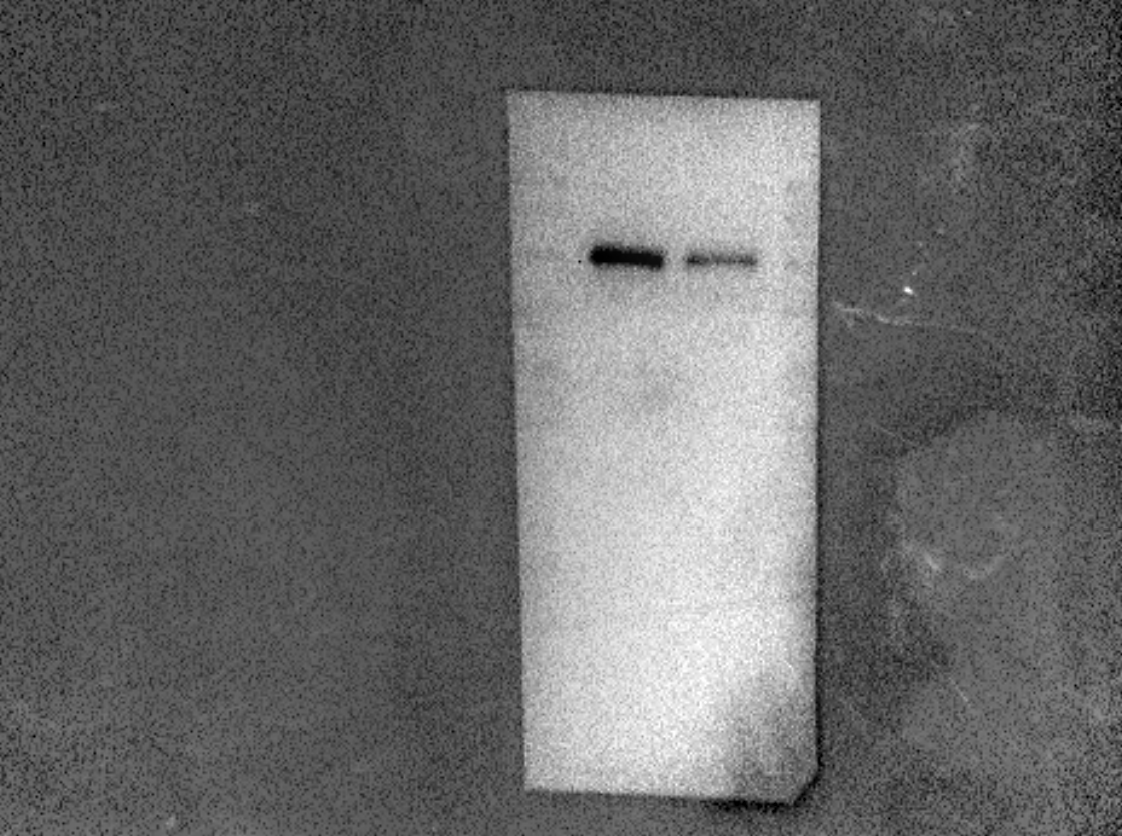


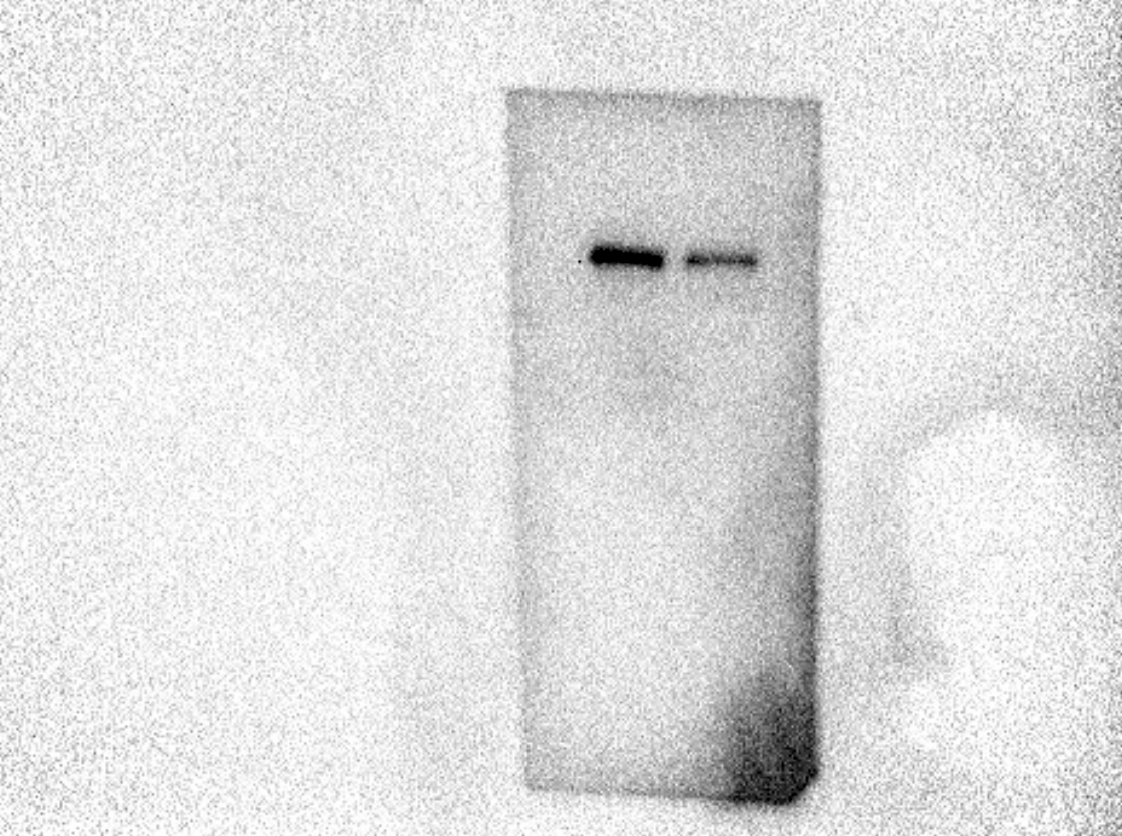


SMAD9-2


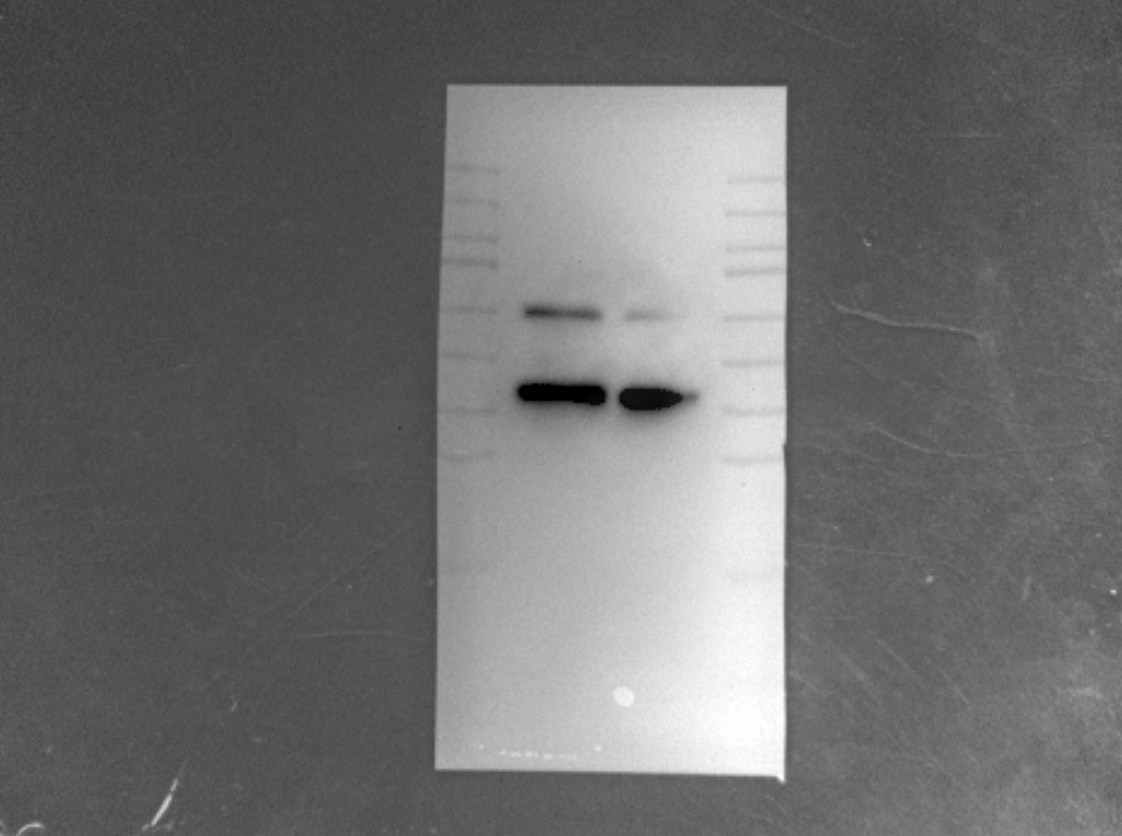


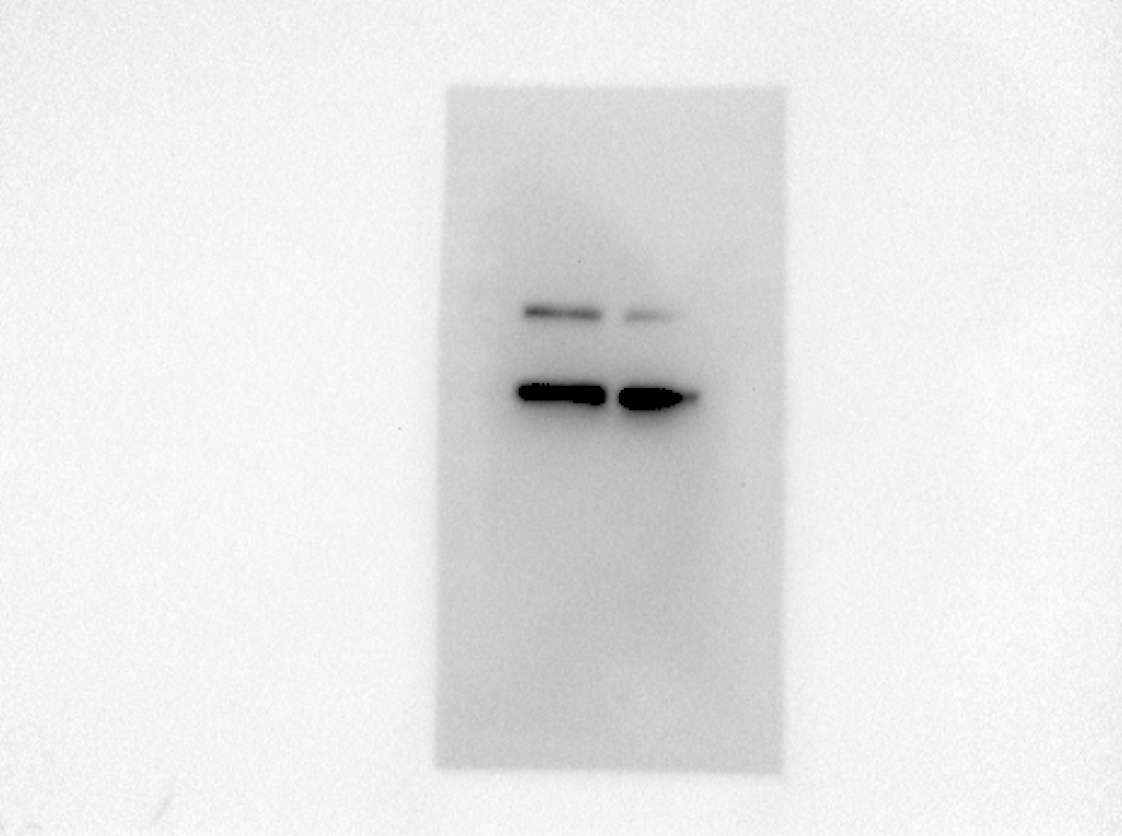


SMAD9-3


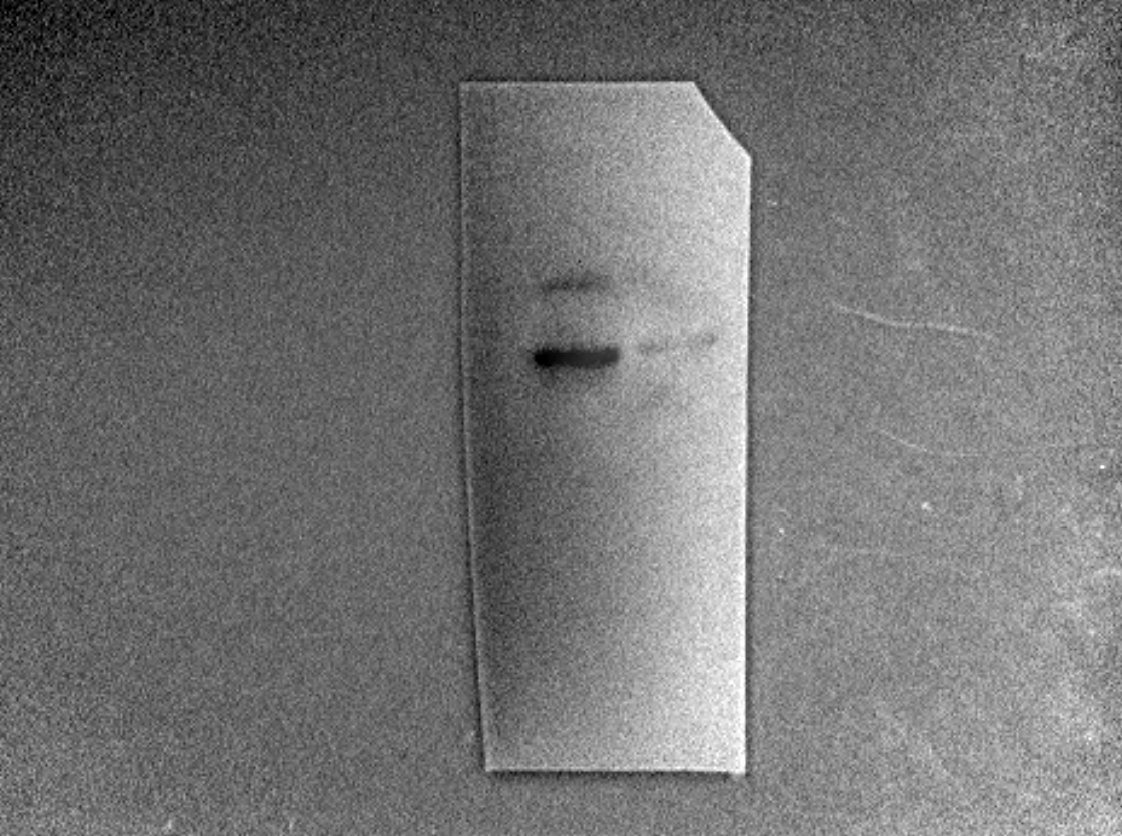


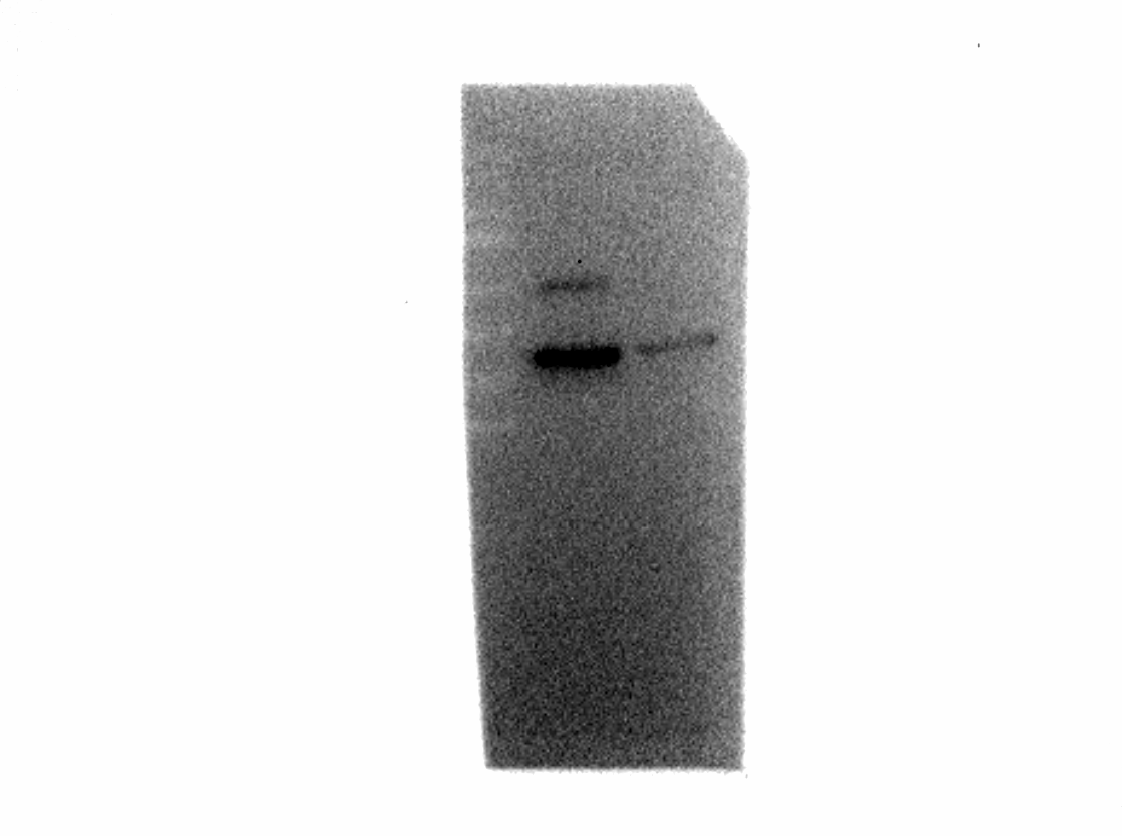

Supplement: Supplementary file 3 — Supplementary Material 3. [file 12672_2025_3240_MOESM3_ESM.docx]
